# Genetic score omics regression and multitrait meta-analysis detect widespread *cis*-regulatory effects shaping bovine complex traits

**DOI:** 10.1093/pnasnexus/pgaf208

**Published:** 2025-07-02

**Authors:** Ruidong Xiang, Lingzhao Fang, Shuli Liu, George E Liu, Albert Tenesa, Yahui Gao, Brett A Mason, Amanda J Chamberlain, Michael E Goddard, CattleGTEx Consortium, Shuli Liu, Yahui Gao, Oriol Canela-Xandri, Sheng Wang, Ying Yu, Wentao Cai, Bingjie Li, Ruidong Xiang, Amanda J Chamberlain, Erola Pairo-Castineira, Kenton D'Mellow, Konrad Rawlik, Charley Xia, Yuelin Yao, Pau Navarro, Dominique Rocha, Xiujin Li, Ze Yan, Congjun Li, Benjamin D Rosen, Curtis P Van Tassell, Paul M Vanraden, Shengli Zhang, Li Ma, John B Cole, George E Liu, Albert Tenesa, Lingzhao Fang

**Affiliations:** Agriculture Victoria, AgriBio, Centre for AgriBiosciences, Bundoora, VIC 3083, Australia; The School of Applied Systems Biology, La Trobe University, Bundoora, VIC 3083, Australia; School of Agriculture, Food and Ecosystem Sciences, The University of Melbourne, Parkville, VIC 3052, Australia; MRC Human Genetics Unit at the Institute of Genetics and Cancer, The University of Edinburgh, Edinburgh EH4 2XU, United Kingdom; Centre of Quantitative Genetics and Genomics, Aarhus University, Aarhus 8000, Denmark; Westlake Laboratory of Life Sciences and Biomedicine, Hangzhou, Zhejiang 310024, China; Animal Genomics and Improvement Laboratory, Henry A. Wallace Beltsville Agricultural Research Center, Agricultural Research Service, USDA, Beltsville, MD 20705, USA; MRC Human Genetics Unit at the Institute of Genetics and Cancer, The University of Edinburgh, Edinburgh EH4 2XU, United Kingdom; The Roslin Institute, Royal (Dick) School of Veterinary Studies, The University of Edinburgh, Midlothian EH25 9RG, United Kingdom; Animal Genomics and Improvement Laboratory, Henry A. Wallace Beltsville Agricultural Research Center, Agricultural Research Service, USDA, Beltsville, MD 20705, USA; Agriculture Victoria, AgriBio, Centre for AgriBiosciences, Bundoora, VIC 3083, Australia; Agriculture Victoria, AgriBio, Centre for AgriBiosciences, Bundoora, VIC 3083, Australia; The School of Applied Systems Biology, La Trobe University, Bundoora, VIC 3083, Australia; Agriculture Victoria, AgriBio, Centre for AgriBiosciences, Bundoora, VIC 3083, Australia; School of Agriculture, Food and Ecosystem Sciences, The University of Melbourne, Parkville, VIC 3052, Australia

## Abstract

To complete the genome-to-phenome map, transcriptome-wide association studies (TWAS) are performed to correlate genetically predicted gene expression with observed phenotypic measurements. However, the relatively small training population assayed with gene expression could limit the accuracy of TWAS. We propose genetic score omics regression (GSOR) correlating observed gene expression with genetically predicted phenotype, i.e. estimated breeding values (EBVs) in agriculture or polygenic score (PGS) in medicine. The score, calculated using variants near genes with assayed expression (*cis*-EBV or *cis*-PGS), provides a powerful association test between *cis-*effects on gene expression and the trait. In simulated and real data, GSOR outperforms TWAS in detecting causal/informative genes. We applied GSOR to transcriptomes of 16 tissues (*N* ∼ 5,000) and 37 traits in ∼120,000 cattle and conducted multitrait meta-analyses of omics-associations (MTAO). We found that, on average, each significant gene expression and splicing mediates *cis*-genetic effects on 8–10 traits. Many prioritized genes by GSOR and MTAO can be verified by Mendelian randomization analysis and show significantly reduced d*N*/d*S*, suggesting elevated evolutionary constraint for these genes. Using multiple methods, we detect expression levels of genes and/or RNA splicing events underlying previously thought single-gene loci to influence multiple traits. For example, the expression and RNA splicing of *DGAT1* from multiple tissues regulated milk production, mastitis, gestation length, temperament, and stature. Also, gene expression and splicing of *ABO* (Histo-blood group) and *ACHE* (acetylcholinesterase, Cartwright blood group) affected protein concentration and mastitis, respectively. Taken together, our work provides new methods and biological insights for prioritizing informative omics–phenotype associations in mammals.

Significance Statement While genome-wide association studies identify many links between DNA variants and complex traits, the genes, cells, or tissues via which DNA variants act are largely unknown, especially in nonhuman species. We developed a method called genetic score omics regression (GSOR) which associates assayed gene expression with genetic score, i.e. predicted phenotype using large reference populations. We also extended GSOR to multitrait analyses and applied it to datasets consisting of >110,000 cattle with 37 complex traits and transcriptome data of ∼5,000 cattle across 16 tissues. Through simulations and validations using causal inferences with real data, GSOR showed competitive performance over traditional methods. Combining GSOR with other analyses identified novel putative regulatory mechanisms behind cattle complex traits and mammalian evolution.

## Introduction

Genome-wide association studies (GWAS) test millions of genome variants, such as single nucleotide polymorphisms (SNPs), for association with quantitative traits. Significant associations map a quantitative trait locus (QTL) to a genomic region tracked by the associated variant. Since most associations involve noncoding genetic variants, gene regulation is expected to mediate the effects of many QTL. While this can be investigated by associating gene expression with complex traits, the two measurements are not always on the same individuals, and this prevents the direct association analysis. However, it is possible to find a genetic association between predicted gene expression and complex traits where the prediction of gene expression is made from SNP genotypes in the individuals with complex trait phenotypes, and the prediction equation is trained in other individuals with SNP genotypes and gene expression measurements. This is commonly referred to as a transcriptome-wide association study (TWAS) (1, 2).

The power of TWAS is determined by the accuracy of predicting gene expression from SNP genotypes and by the proportion of phenotypic variance explained by the expression of a single gene. Most datasets with gene expression measurements are not large, and the prediction of gene expression is often limited to *cis*-eQTL because *trans* effects are small and so hard to estimate accurately. Also, given the polygenicity of most complex traits, the predicted expression based on *cis*-eQTL of a single gene is likely to explain only a small proportion of the variance of a complex trait.

In this study, we propose an alternative approach to estimating the genetic association between gene expression and complex traits which we call genetic score omics regression (GSOR). The datasets with complex trait measurements and SNP genotypes are often very large and can be used to train a prediction equation that predicts complex trait genetic values from SNP genotypes. The prediction is called estimated breeding value (EBV) in animals and plants or a polygenic score (PGS) in humans (3). Then, this prediction equation can be applied to individuals with actual gene expression measurements to correlate gene expression with EBV or PGS. Potentially, GSOR has two advantages over traditional TWAS. Firstly, the prediction of EBV/PGS should be more accurate because it is trained on a much larger dataset than the prediction of gene expression. Secondly, the part of the EBV/PGS due to effects of SNPs close to the gene, i.e. the local or *cis* EBV (4, 5)/PGS, can be calculated and correlated with gene expression. These SNPs are also those responsible for *cis*-eQTL, so the test for a correlation between *cis* effects on gene expression and complex trait EBV is more powerful than in TWAS. The above description of GSOR assumed the use of gene expression measurements, but it could be applied to any omics phenotype. Here, we use gene expression together with RNA splicing.

GWAS are often followed by a meta-analysis of the effect (beta and SD or SE) of variants. Where GWAS summary statistics are available for several traits, a multitrait meta-analysis of GWAS can be used to identify variants affecting multiple traits (6), i.e. pleiotropy. Similarly, TWAS or GSOR also produces association summary statistics between gene expression and multiple phenotypes. Therefore, a multitrait meta-analysis can also be applied to such summary data to investigate the pleiotropic effects mediated by regulatory mechanisms.

Understanding causal mechanisms behind QTL is important but challenging. In humans, large-scale GWAS of both conventional and molecular phenotypes, such as gene expression (7) and RNA splicing (8), improved the understanding of QTL causal effects. In animals, only a few causal QTL are identified, and one of the most extraordinary QTL is a mutation in the gene for diacylglycerol *O*-acyltransferase 1 (*DGAT1*) in cattle. This single QTL explains 30–40% of the phenotypic variance of milk production traits (9, 10). While this QTL was previously identified to be caused by a protein-coding mutation (9, 11, 12), more recent studies indicated regulatory effects (10, 13), possibly due to multiple causal mutations. The new CattleGTEx (14) and the FAANG consortium (15) provide opportunities to explore the causal regulatory mechanisms behind this QTL.

A complication with the interpretation of GWAS and TWAS results is caused by linkage disequilibrium (LD). One SNP may be associated with the expression of a gene and with a complex trait because of LD between this SNP and both a QTL for the trait and an eQTL for gene expression. However, if all SNPs that affect the expression of the gene have a proportional effect on the complex trait, then this is evidence that the gene expression causes variation in the complex trait. This is the logic of Mendelian randomization as implemented in summary-data-based Mendelian randomization (SMR) (16), which we use here to validate our results and explore causality. In addition, genes with important functions in mammals may have undergone purifying selection or are under evolutionary constraints across species. In this paper, we also investigate whether prioritized putatively causal genes show evidence of purifying selection.

We developed and applied GSOR, an association-based test, to transcriptomes from 16 tissues from ∼5,000 cattle and 37 complex phenotypes from 113,000 cattle to dissect the genetic effects on complex traits mediated by the transcriptome. We propose a meta-analysis to quantify the pleiotropic effects of regulatory loci. After association analyses using GSOR, we then use Mendelian randomization and the ratio of nonsynonymous substitution (d*N*) to synonymous substitution rates (d*S*) (17) to verify these effects and combine GSOR and SMR to dissect regulatory mechanisms behind complex traits. We show that blood group genes *ABO* and *ACHE* (Cartwright blood group) mediate regulatory effects on protein concentration and mastitis via expression and splicing, supporting conserved and widespread regulatory effects on mammalian complex traits.

## Results

### Genetic score omics regression

**Fig. 1.**
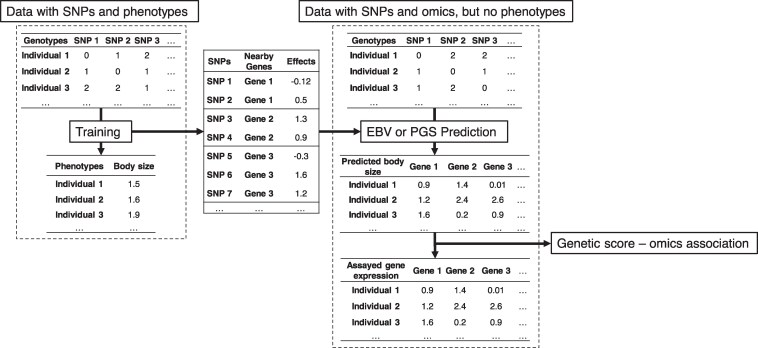
Graphic illustration of the workflow of GSOR. The left panel illustrates the dataset with SNPs and phenotypes where the association analysis (model training) between SNP and phenotypes, e.g. body size, can be conducted. This will deliver effects (models, middle panel) on the body size of each SNP which can be linked to genes based on their distance to genes. For example, SNPs had ±1 Mb to genes were *cis*. Using such effects, in a new dataset where there are SNPs and omics but no phenotypes (right panel), one can use *cis*-SNP effects to compute predicted local EBV (*cis*-EBV) or PGS (*cis*-PGS). Because SNPs are annotated to genes, then one can compute as many profiles of local or *cis* EBVs/PGSs for body size as the number of genes. As there are assayed gene expressions in the new dataset, one can associate local or *cis* EBVs or PGSs with assayed gene expression, which is the last step for GSOR. The principle applies to *trans* effects and genome-wide effects as well, where SNPs far from the gene or genome-wide are used for calculation of EBVs or PGSs.

It is more likely for a population with phenotypic records to have a larger sample size than a population with omics datasets, such as gene expression and RNA splicing. Therefore, we developed GSOR which is an association test to estimate the effects (*b*) of omics features (Ω) on a complex phenotype leading to an EBV or PGS, ${\hat{g}}_{P}$ (Fig. 1). We also illustrate the equivalence of the effective testing of associations between TWAS and GSOR in Note S1. However, the advantage of GSOR is the computation of local or *cis*-EBV/PGS using larger training data (see details below and Brief Methods). The basic form of GSOR is:


(1-3) $$\{\begin{array}{c} \;{\hat{g}}_{P_{cis}}=\Omega b_{cis}+e\;\;\;\;\;\;\;\;\\ {\hat{g}}_{P_{trans}}=\Omega b_{trans}+e\\ {\hat{g}}_{P_{\mathrm{total}}}=\Omega b_{\mathrm{total}}+e\;\; \end{array}$$

The response variable is the EBV or PGS for a complex trait (${\hat{g}}_{P}$) estimated using either genetic variants close (±1 Mb of transcription start site [TSS]) to a gene (${\hat{g}}_{P_{cis}}$) or all other genetic variants (${\hat{g}}_{P_{trans}}$), or is the total EBV/PGS ${\hat{g}}_{P_{\mathrm{total}}}={\hat{g}}_{P_{cis}}+{\hat{g}}_{P_{trans}}$. Accordingly, $b_{cis}$, $b_{trans}$, and $b_{\mathrm{total}}$ is the coefficient of regression of ${\hat{g}}_{P_{cis}}$, ${\hat{g}}_{P_{trans}}$, and ${\hat{g}}_{P_{\mathrm{total}}}$, on the gene expression or splicing value. Ω is an *n* × 1 vector of omics values, such as gene expression or RNA splicing corrected for other fixed effects, such as breed, sex, and experiments. GSOR allows the fitting of random effects of a relationship matrix to control for population structures or confounding factors. GSOR is freely available at https://github.com/rxiangr/GSOR-and-MTAO.git.

To compare GSOR with the conventional TWAS, we analyzed simulated and real data of 113,000 cattle with 16M sequence genotypes and 37 complex trait phenotypes and 945 cattle with 6M sequence genotypes and gene expression in blood (see Brief Methods). To match the implementation of GSOR, TWAS was also conducted using linear mixed models where gene expression predictors were trained by jointly fitting two genomic relationship matrices (GRMs) built using *cis* and *trans* variants. The predicted gene expression was then correlated with the complex trait phenotypes.

**Fig. 2.**
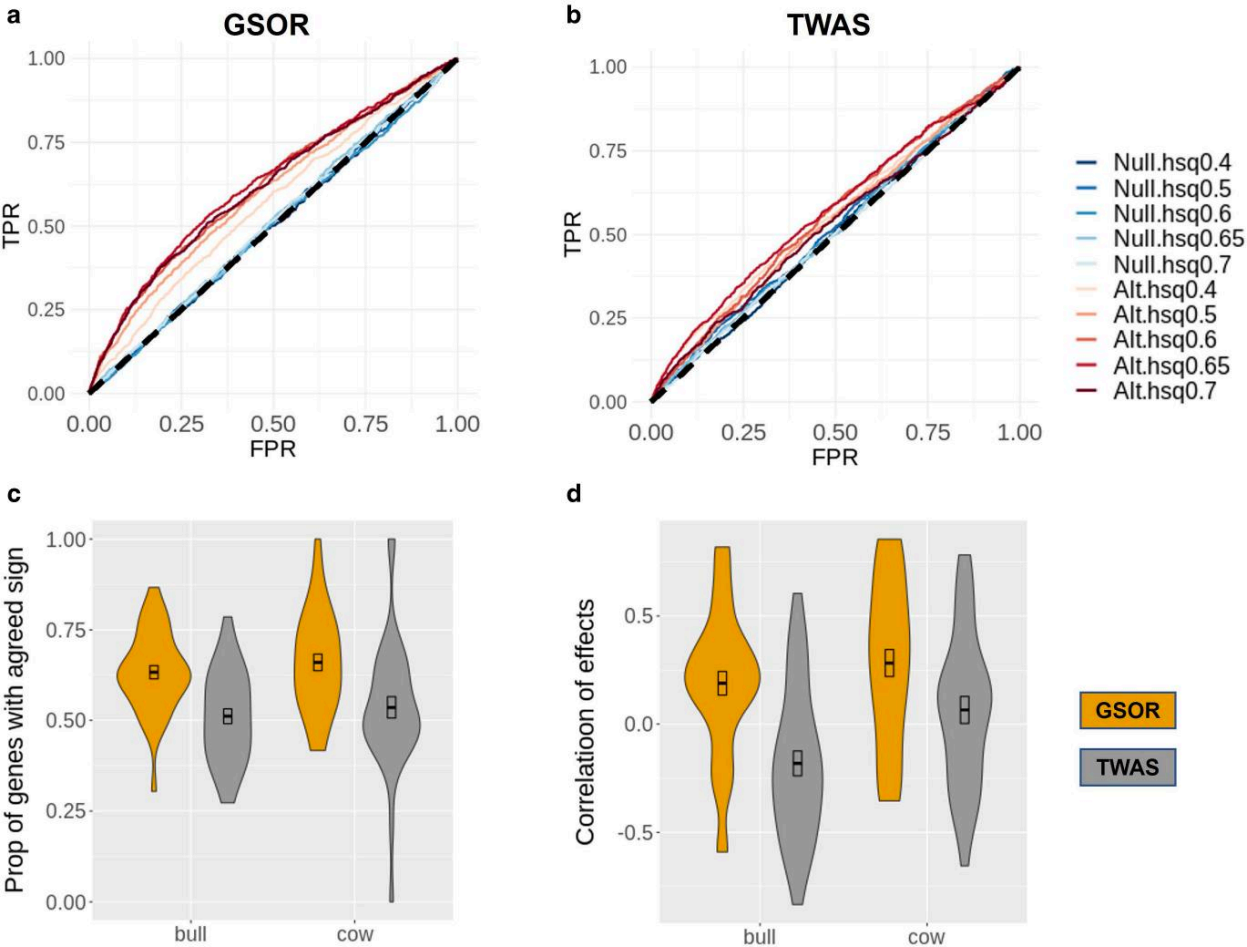
Comparison of results between GSOR and TWAS using simulations and real data. ROC analysis of results from GSOR and TWAS using simulated data are shown in (a) and (b), respectively. Ten scenarios were simulated with varying heritability (hsq) of traits. Five traits were simulated under the null (Null) where no causal eQTL overlapped with causal QTL and another five traits were simulated under the alternative (Alt) scenarios where causal eQTL overlapped with causal QTL for >1,000 genes. A comparison of results from real data is shown as violin plots in (c) and (d). In bull and cow datasets, the agreement of gene expression–phenotype association between *cis*- and *trans*-predicted values were compared. The comparison was based on the proportion of genes with the same sign (c) or correlation of effects (d), between *cis-* and *trans*-predicted values for 37 traits in each sex. TPR, true positive rate; FPR, false-positive rate.

Using real bovine genotype data (*N* = 102K) and ARS-UCD1.2 genome coordinates, we simulated causal *cis* and *trans* eQTL for 16,600 genes and created five scenarios of nulls where causal eQTL and causal QTL did not overlap and five alternative scenarios where causal eQTL and causal QTL did overlap (see Brief Methods). Using receiver operating characteristic (ROC) analysis of results, we showed that GSOR outperformed TWAS in detecting causal genes based on *cis*-predicted gene expression (Fig. 2a and b) and *cis*  *+*  *trans*-predicted gene expression (Fig. S1a and b, Table S2). In the current simulation framework, neither GSOR nor TWAS had the power to detect causal genes based on *trans*-predicted gene expression (Fig. S1c and d, Table S2).

We next compared the results of GSOR and TWAS by analyzing real blood gene expression data and 37 traits of 113,000 bulls and cows (Table S1). If the increased expression of a gene causes a change in a complex trait, we expect the direction of that change to be the same for both *cis* and *trans* effects on gene expression. In other words, the direction of the association between the genetic component of the gene expression and the phenotype should be independent of how the expression is predicted. Using simulated data, we show that this was true for most genes that were significantly associated with phenotypes (Fig. S2). We observed that, in both sexes across 37 traits, the agreement of the direction of *cis* and *trans* effects and the correlation of effects between them was higher in GSOR than in TWAS (Fig. 2c and d). In addition, we estimated the $\pi_{1}$ value (lower bound on the proportion of truly alternative features (18), commonly used to indicate the proportion of replicated associations between different analyses (7, 14)) for results of GSOR and TWAS. We observed relatively higher $\pi_{1}$ for GSOR than TWAS when replicating the gene expression–phenotype between *cis-* and *trans*-predicted $\hat{g}$ (Fig. S3a), although for both GSOR and TWAS, $\pi_{1}$ between *cis-* and *trans*-predicted $\hat{g}$ was low. We also replicated the gene expression–phenotype association between bulls and cows, where we observed a much higher $\pi_{1}$ for GSOR than TWAS using *cis*-predicted $\hat{g}$ (Fig. S3b). Overall, our results support the conclusion that GSOR has advantages over conventional TWAS in detecting genes whose expression is causally associated with complex traits.

To test whether the assumption of GSOR is influenced by the causal direction, we performed additional simulations where besides SNPs causing gene expression levels, gene expression causes phenotypes. The analyses of these simulated data showed that GSOR is at least as powerful as TWAS (Fig. S4). In the *cis* + *trans* analysis, GSOR performed slightly better than TWAS with suggestive significance (*P* = 0.09; Fig. S4). Therefore, these results support that the assumption of GSOR holds when the direction of causality is specified. One of the most popular models for conducting TWAS is PrediXcan (2) which implemented an elastic net model (ELnet) to train and predict *cis*-gene expression rather than using genomic best linear unbiased prediction (gBLUP). By the analysis of simulated data, we found that *cis*-GSOR outperformed *cis*-TWAS-ELnet (Fig. S5). We also counted the number of causal genes detected by GSOR, *cis*-TWAS-gBLUP and *cis*-TWAS-ELnet and we found that GSOR detected the highest proportion of causal genes, and the performances between *cis*-TWAS-gBLUP and *cis*-TWAS-ELnet were not significantly different (Table S3).

### Multitrait meta-analysis of omics-associations

**Fig. 3.**
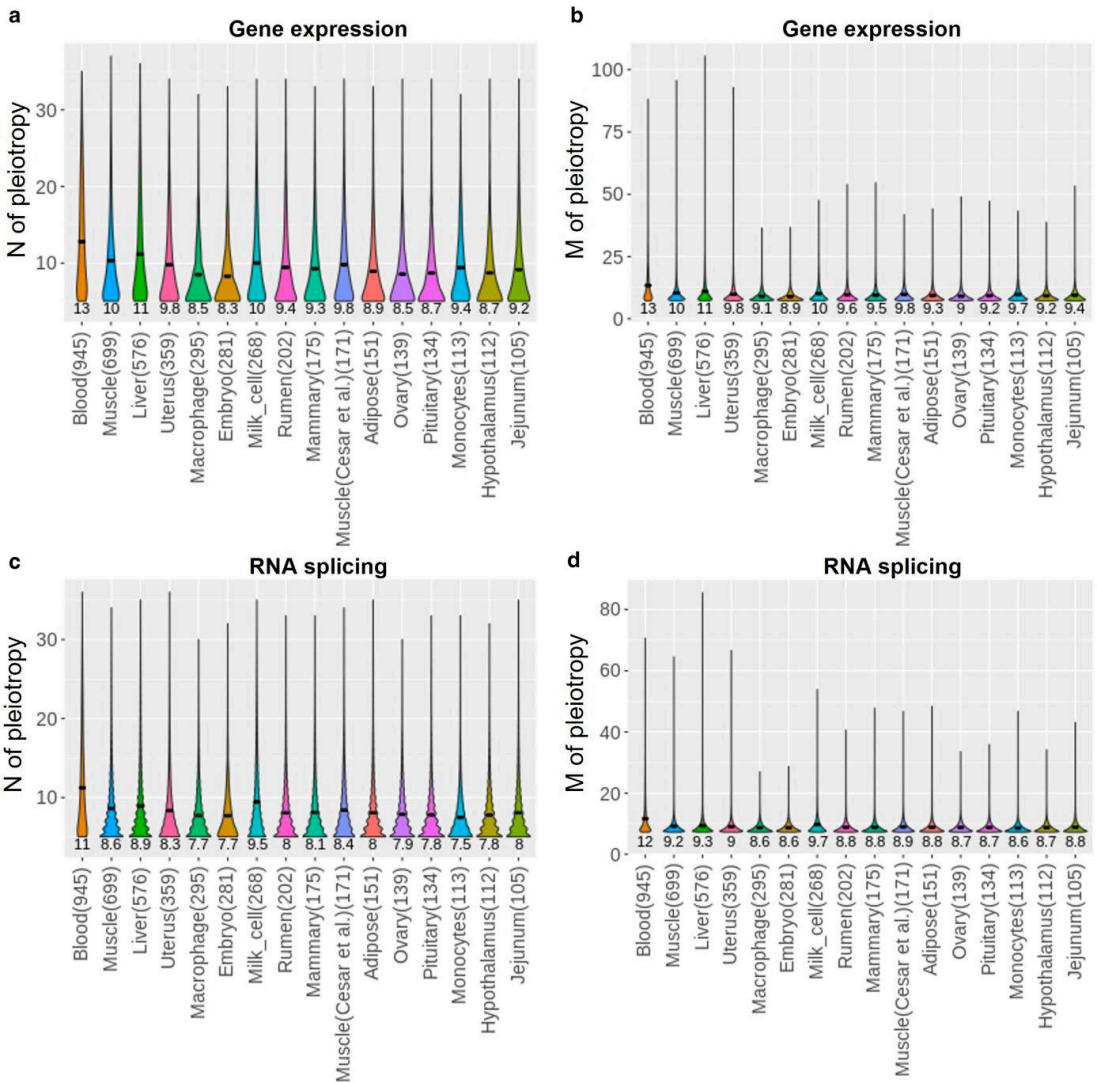
Gene expression and RNA splicing mediated *cis* pleiotropy. The average number of traits (*N*) associated with a gene expression (a) and the average magnitude (*M*) of pleiotropy with a gene expression (b); and the average *N* associated with a splicing event (c) and the average *M* of pleiotropy with a splicing event (d) are shown across 16 tissues. The numbers under each plot are the average values of *N* and *M* per tissue. The numbers in brackets are the sample size of each tissue.

We applied GSOR to transcriptome data (gene expression and RNA splicing) of 16 tissues, combining newly generated data with data from CattleGTEx V0 (14) with a sample size >100 (Table S4) and ${\hat{g}}_{P}$ of 37 cow traits (see Brief Methods). Summary statistics of GSOR on gene expression and RNA splicing from 16 tissues across 37 traits of 103K cows are publicly available at https://figshare.com/s/c10ffab5abf329b1318f. The average genomic inflation lambda of GSOR analyses across tissues and traits is 1.09 (SD = 0.27; Fig. S6). To gain novel insights from vast summary statistics from GSOR, we introduce a multitrait meta-analysis of omics-associations (MTAO) to quantify the extent of *cis*-pleiotropy mediated by omics. MTAO estimates two statistics for each omics feature (including gene expression or splicing events) from each tissue: (i) the number of traits affected ($N_{\mathrm{pleio}}$) and (ii) the magnitude of multitrait effects ($M_{\mathrm{pleio}}$). The estimation of $N_{\mathrm{pleio}}$ adopted the method from Jordan et al. (19) with increased rigor of significance testing (see Brief Methods). The estimation of $M_{\mathrm{pleio}}$ models the χ^2^ distribution of signed *t*-values of each omic feature along with the correlation matrix of *t*-values across 37 traits to approximate the error covariance matrix (see Brief Methods). The R implementation of MTAO is publicly available at https://github.com/rxiangr/GSOR-and-MTAO/blob/main/README_MTAO.md. MTAO revealed that gene expression and RNA splicing mediate widespread *cis*-pleiotropic effects on complex traits (Fig. 3a and b). Across 16 tissues and 37 traits based on 3,612 (SD = 1,037) significant genes (Table S3), on average, the gene expression mediated *cis*-pleiotropic effects on 9.7 traits (ranging from 8 to 13) with an average magnitude of 9.8 (ranging from 9 to 13). Based on 23,477 (SD = 10,633) significant introns (Table S5), on average, splicing of an intron mediated *cis*-pleiotropic effects on 8.4 traits (ranging from 8 to 11) with an average magnitude of 9.1 (ranging from 9 to 12; Fig. 3c and d).

### MTAO, Mendelian randomization, and selection

**Fig. 4.**
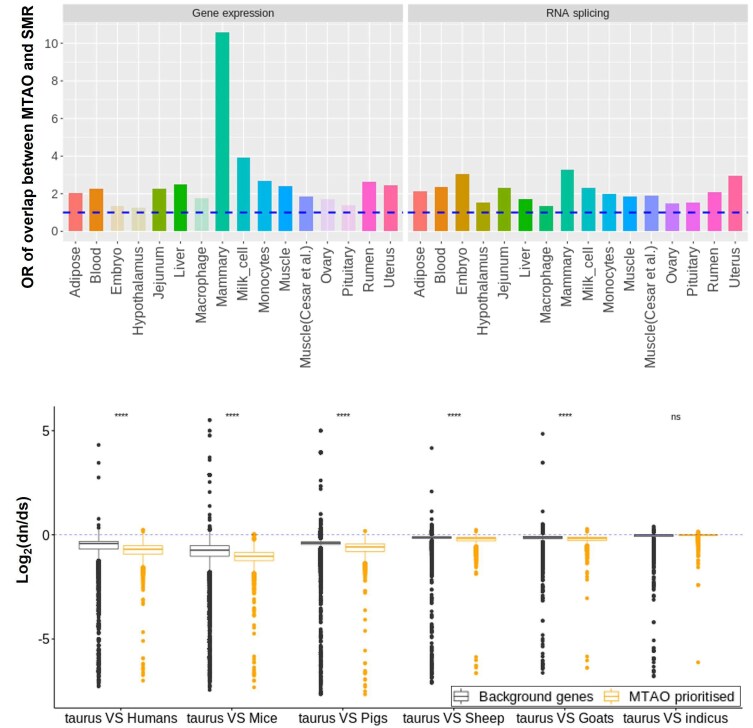
Supportive evidence for MTAO. a) Overlap of prioritized genes/introns between MTAO and multitrait SMR. Bars represent the odds ratio (OR) of Fisher's exact test of the overlap. The dashed blue line indicates OR = 1. Bars with transparent colors indicate the *P*-value of Fisher's exact test >0.05 after multitest adjustment (embryo, hypothalamus, macrophage, ovary, and pituitary in gene expression). b) Nonsynonymous (d*N*) to synonymous substitution rate (d*S*) ratios for MTAO-prioritized genes compared between *B. taurus taurus* cattle (taurus) and other mammals (1-to-1 orthologs), including *Bos indicus* which is a subspecies of cattle. ****t test, *P* < 1 × 10^−4^; ns: *t* test not significant. In total, 14,504 ortholog genes participated in the analysis.

The association between expression and splicing with traits could be due to LD between eQTL or sQTL and QTL for complex traits. To test whether variation in gene expression or splicing causes variation in complex traits, we conducted the SMR in combination with the heterogeneity in dependent instruments (HEIDIs) (16) test based on *cis*-eQTL and sQTL mapped from 16 tissues and GWAS of 37 traits (20, 21) (see Brief Methods). Where results of SMR are available for multiple traits for a gene or an intron that passed the HEIDI test, we also used a multitrait meta-analysis to combine SMR results across traits to identify genes or splicing events causing variation in more than one trait (see Brief Methods). Then, we compared the genes/spliced introns prioritized by MTAO and by multitrait SMR to check the extent of overlap (Fig. 4a, Table S6). Fisher's exact tests show that the overlap of prioritized genes/spliced introns between MTAO and SMR is on average 2.4 times more than expected by random chance and is significant in most tissues (Fig. 4a). In addition, we also analyzed the pathway enrichment for genes detected by GSOR and SMR. We found that genes detected by both GSOR and SMR had more significantly enriched pathways than SMR alone (Fig. S7, Tables S7 and S8).

In addition, based on 1-to-1 orthology, we compared the d*N*/d*S* ratios of genes prioritized by MTAO between cattle and other species, including humans, mice, and *Bos taurus indicus* which is a subspecies closely related to *B. taurus taurus* cattle (Fig. 4b). When comparing cattle and other species, MTAO-prioritized genes showed significantly reduced d*N*/d*S* ratios than random genes.

### Exploring trait-relevant tissues

**Fig. 5.**
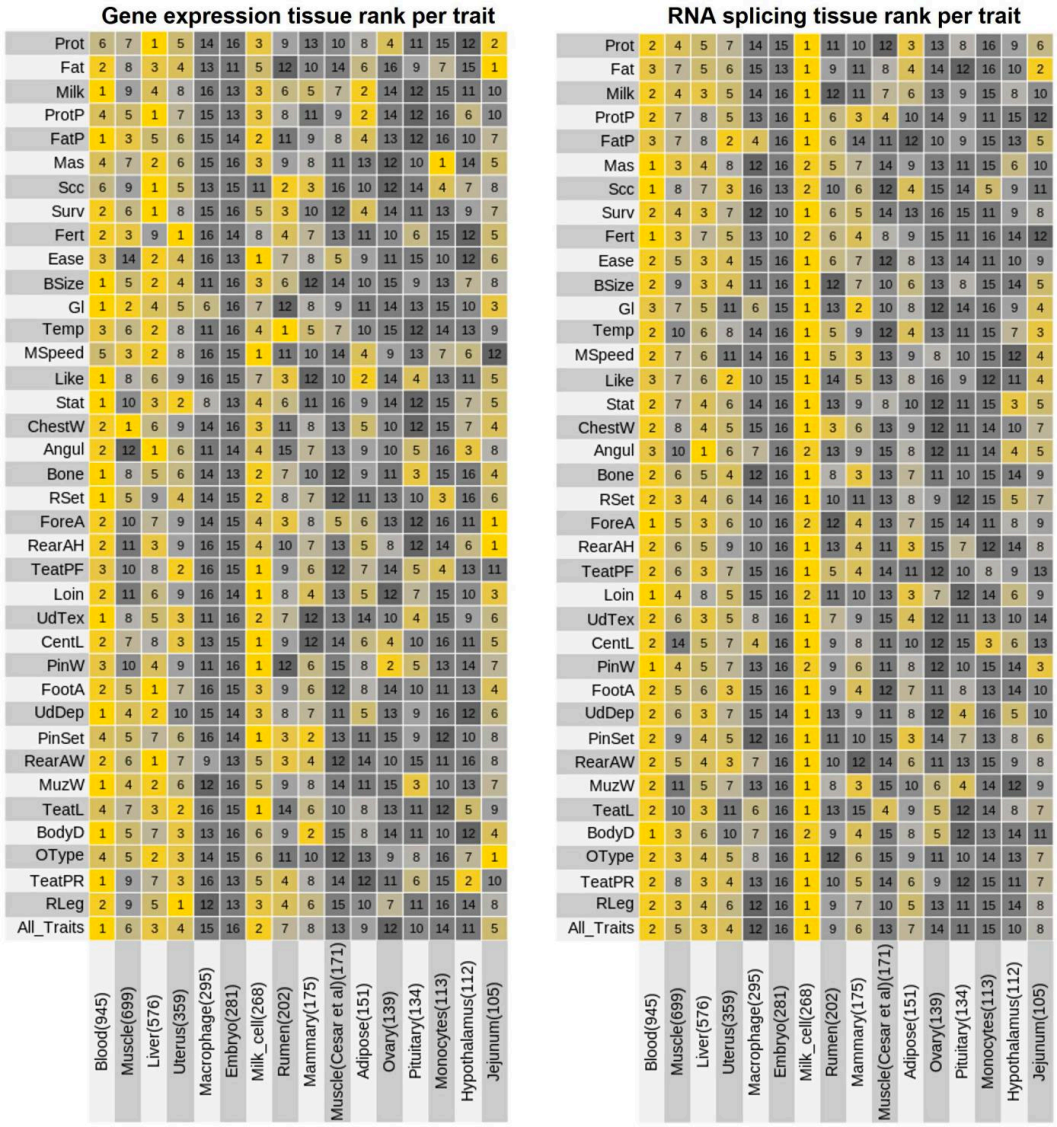
Ranking of tissues based on their importance to each trait. For each trait, we estimated the sum of the effects of GSOR and SMR across genes (or introns) per tissue adjusted by the number of genes and individuals. This sum was used to rank tissues for each trait. The numbers within cells indicate the tissue ranging from 1 to 16 per trait. The ranking for “All_Traits” is the ranking of tissues averaged across all traits. The numbers in brackets are the sample size of each tissue.

We next used results obtained from GSOR and SMR together to rank tissues according to their relationship with each trait. We used a heuristic index that combined results from GSOR, SMR, and HEIDI with the number of genes and individuals to rank tissues for each trait (see Brief Methods; Fig. 5). Our explorative analysis shows that blood, milk cells, liver, and uterus had the highest and most consistent ranking in their connections with the traits analyzed. The ranking of tissues on average had a low correlation (Spearman’s *rho* = 0.35) with the sample size, and these correlations were not significant (Table S9).

### Regulatory mechanisms underlie previously thought single-gene loci

**Fig. 6.**
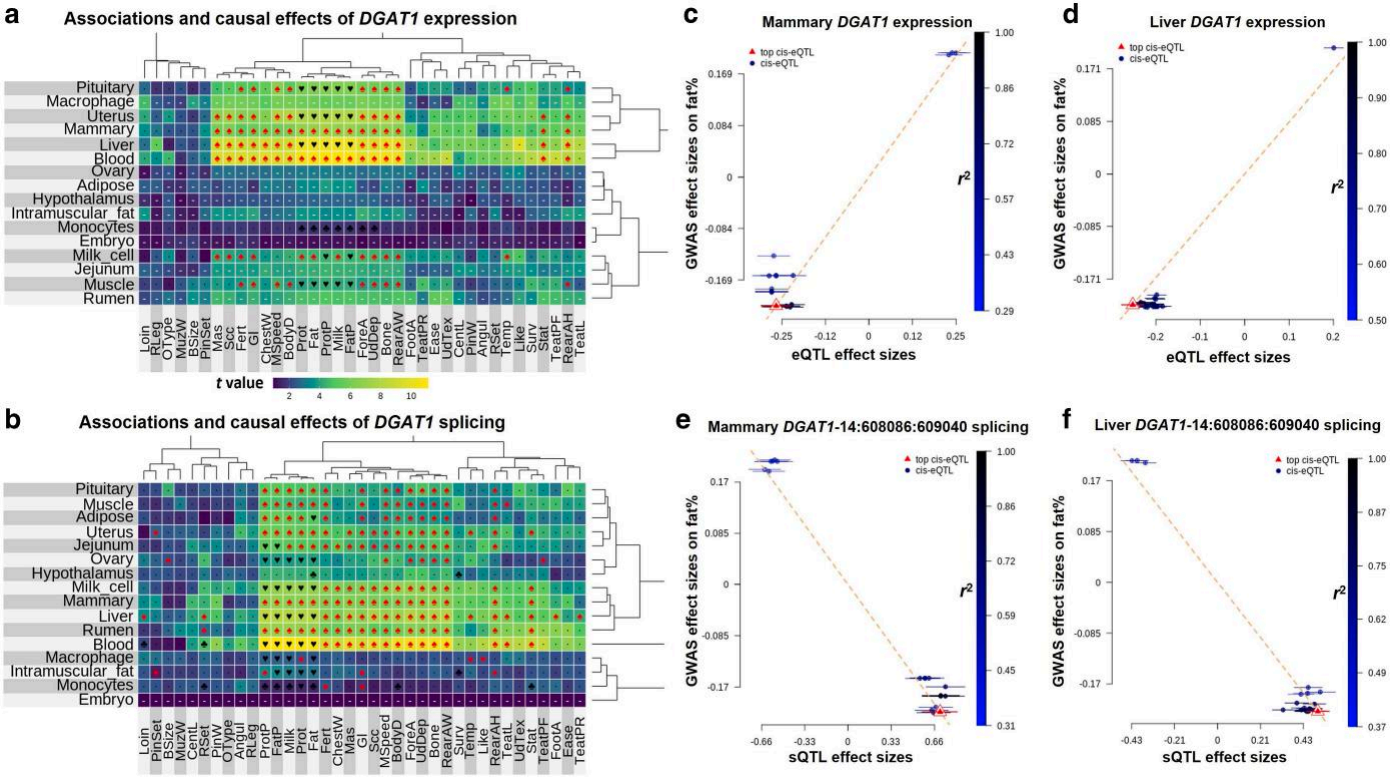
Widespread regulatory effects of *DGAT1*. The heat map of effects of *DGAT1* expression (a) and splicing (b) across tissues and traits based on GSOR. In these heat maps, spades indicate regulatory effects inferred using SMR independent of LD; hearts indicate the regulatory effects confounded by LD while clubs indicate regulatory effects without testing LD due to not enough SNPs. Dots represent insignificant SMR test and white hyphens indicate no e/sQTL or QTL could be used for the SMR test. The dendrogram represents the hierarchical clustering of effects. The color scale of heat maps is based on the magnitude of *t* (beta/SE) value of GSOR. c and d) Examples of Mendelian randomization using the expression eQTL and GWAS of fat%. e and f) Examples of Mendelian randomization using splicing sQTL and GWAS of fat%. 14:608086-609040 indicates the location of the spliced intron in *DGAT1*. Note that in c–f, according to the method (16), the estimate of bxy was at the top *cis*-eQTL (rather than the regression line).

For the first time, we provide statistical evidence to support that gene expression and RNA splicing at *DGAT1* regulate many traits in many tissues, and such regulatory links are not restricted to milk production traits (Fig. 6a and b). Both MTAO and SMR found putative phenotypic effects of *DGAT1* expression and/or splicing in blood, liver, mammary gland, milk cells, and uterus (Fig. 6a–e). *DGAT1* expression and splicing affected milk production, mastitis (MAS, average correlation with milk production traits ${\bar{r}}_{\mathrm{milk}}$ = −0.01), gestation length (Gl, ${\bar{r}}_{\mathrm{milk}}$ = −0.03), temperament (Temp, ${\bar{r}}_{\mathrm{milk}}$ = −0.04), and stature (Stat, ${\bar{r}}_{\mathrm{milk}}$ = 0.09). Our results show that RNA splicing of *IGF2* (insulin-like growth factor 2) (22) affects many traits in tissues of liver and adipose (Fig. S8). Also, the expression of *MGST1* (microsomal glutathione *S*-transferase 1) (23) affects milk production traits in milk cells and the hypothalamus (Fig. S9). In addition, we did not observe regulatory effects from *GHR* (growth hormone receptor; Fig. S10) with reported missense mutations (24) at sites conserved across vertebrates (25).

We identified many new or unannotated loci, including two blood group genes, *ABO* (Histo-blood group) and *ACHE* (acetylcholinesterase, Cartwright blood group), affected protein concentration and mastitis, respectively, via both gene expression and splicing in blood (Table 1 and Data S1). Also, *DCXR* (dicarbonyl and L-xylulose reductase), which plays a significant role in glucose metabolism and causes human pentosuria (26) is linked to cattle fat yield via both gene expression and splicing in blood. *FLII* (FLII actin remodeling protein) affected cattle gestation length via splicing in milk cells. It is a gene related to embryogenesis in *Drosophila* (27). Transcription factor gene *TAF9* (TATA-box-binding protein–associated factor 9) affects cattle milk speed via splicing in blood.

**Table 1.** Summary of links to cattle traits via gene regulation prioritized by GSOR (*P*-value shown in P_GSOR_), SMR (*P*-value shown in *P*_SMR_ and *P*_HEIDI_), and MR-PRESSOR.

Gene	Name	Chr	Start	End	Tissues	Traits	*P*_GSOR_	*P*_SMR_	*P*_HEIDI_	*P*_MR-PRESSO_
ENSBTAG00000018804	*CELSR2*	3	34,131,464	34,157,519	Liver	ProtP	7E−08	2E−07	0.1484	3.45E−68
ENSBTAG00000001136	*ELAPOR1*	3	34,200,677	34,215,659	Milk_cell	ProtP	3E−07	4E−05	0.8247	1.0E−300
ENSBTAG00000014655	*MYO1A*	5	56,378,741	56,406,111	Blood, Liver	Ease	3E−12	1E−05	0.0720	2.8E−84 (Blood ns)
ENSBTAG00000008541	*MGST1*	5	93,497,064	93,521,047	Milk_cell	Prot, Fat, Milk, ProtP, FatP	1E−11	9E−17	0.8240	1.7E−110
ENSBTAG00000007556	*DYRK4*	5	105,528,525	105,552,494	Blood	Stat	2E−05	4E−07	0.3218	6.8E−305
ENSBTAG00000054859	Unknown	5	116,545,881	116,552,846	Muscle	ProtP	3E−15	7E−07	0.1392	3.6E−34
ENSBTAG00000020893	*MATN3*	11	78,828,218	78,841,622	Blood, Liver	Stat	4E−64	8E−07	0.0606	3.1E−34
ENSBTAG00000049030	Unknown	11	104,152,769	104,157,310	Blood	FatP	3E−22	1E−06	0.2623	3.1E−239
ENSBTAG00000012525	*ABO*	11	104,176,840	104,214,809	Jejunum	ProtP	2E−07	1E−05	0.9831	na
ENSBTAG00000012353	*ZNF34*	14	309,282	313,478	Blood	Prot, Fat, Milk, ProtP, FatP, Gl	3E−10	2E−14	0.2054	1.2E−141
ENSBTAG00000026356	*DGAT1*	14	603,813	612,791	Blood, Mammary	Prot, Fat, Milk, ProtP, FatP, Gl	1E−26	2E−28	0.1188	2.7E−211
ENSBTAG00000048486	*IQANK1*	14	1,006,976	1,027,840	Milk_cell, Muscle, Liver	Prot, Fat, Milk, ProtP, FatP	3E−07	8E−20	0.0775	7.5E−61 (Milk_cell ns)
ENSBTAG00000005762	*LYNX1*	14	1,669,738	1,672,645	Milk_cell, Blood, Muscle	Prot, Fat, ProtP, FatP	7E−09	8E−19	0.1106	2.1E−59
ENSBTAG00000032544	*SPAG1*	14	64,174,147	64,208,187	Blood	Milk, ProtP	5E−12	2E−08	0.5436	6.8E−106
ENSBTAG00000001151	*APLP1*	18	46,566,807	46,576,567	Blood, Milk_cell	Fert	8E−22	3E−05	0.5404	na
ENSBTAG00000037537	Unknown	18	57,136,169	57,143,497	Blood	Fert, BSize, Stat	6E−15	5E−09	0.0961	2.0E−43
ENSBTAG00000008852	*SIGLEC10*	18	57,462,227	57,469,952	Milk_cell	Gl	1E−05	1E−06	0.9951	1.9E−23
ENSBTAG00000000336	Unknown	18	61,158,271	61,171,888	Blood	Gl	3E−06	8E−08	0.8595	3.2E−137
ENSBTAG00000009171	Unknown	18	61,180,951	61,189,526	Blood	Gl	6E−13	1E−10	0.7494	2.5E−114
ENSBTAG00000054918	Unknown	18	61,272,049	61,273,471	Blood	Gl	3E−12	3E−06	0.1134	1.0E−300
ENSBTAG00000045795	*KIR2DS1*	18	62,754,722	62,761,536	Blood	Gl	4E−09	2E−10	0.4330	1.6E−90
ENSBTAG00000046944	Unknown	19	14,327,135	14,327,967	Milk_cell	Ease	2E−06	3E−06	0.8298	na
ENSBTAG00000051950	Unknown	19	27,824,421	27,825,794	Liver, Uterus	Gl	3E−07	1E−06	0.4830	2.9E−75
ENSBTAG00000052414	*HAP1*	19	41,934,418	41,945,539	Blood	MSpeed	2E−40	2E−05	0.8971	1.0E−300
ENSBTAG00000051698	Unknown	19	50,859,602	50,861,369	Blood, Mammary, Uterus	Fat, FatP	6E−37	8E−08	0.0905	2.0E−168
ENSBTAG00000008747	*DCXR*	19	50,873,199	50,876,290	Blood	Fat	8E−11	2E−06	0.3317	1.6E−260
ENSBTAG00000053909	*NAT9*	19	56,648,056	56,652,832	Blood	Gl	6E−10	1E−05	0.3506	1.0E−300
ENSBTAG00000052397	Unknown	25	2,502,513	2,506,851	Muscle	Scc	2E−06	5E−06	0.2807	4.9E−80
ENSBTAG00000001139	*ACHE*	25	35,762,361	35,768,977	Blood	Mas	2E−40	4E−09	0.5087	na
ENSBTAG00000001131	*SLC12A9*	25	35,789,614	35,799,221	Blood	Mas	6E−07	4E−06	0.1626	na
ENSBTAG00000012668	Unknown	25	36,317,416	36,319,388	Milk_cell	Mas	3E−12	2E−06	1.0000	1.0E−300

*P*-value shown in *P*_MR-PRESSO_. Note that *P*_HEIDI_ < 0.05 indicates LD confounding. ns, not significant; na, analysis did not converge.

## Discussion

The current study shows that GSOR, an association test, has advantages over TWAS in finding genes whose expression or splicing is associated with complex traits. Many methods can be used to detect the association between gene expression and complex traits (28–31). There is also a method called mixOmics (32) which conducts an explorative analysis of multiomics data without incorporating genetic information. However, one of the advantages of GSOR is due to the use of the *cis*-EBV or *cis*-PGS, which can be obtained by training using a large sample size for complex traits. The ability to calculate a *cis*-genetic score that is local to the gene whose expression is being considered leads to a more powerful test for *cis*-eQTL or sQTL effects. This is a key difference and advantage of GSOR compared with TWAS. In many TWAS analyses, only *cis* effects were mapped, and we have also attempted to map *trans* effects using GSOR which again highlights the difficulty of such analysis. Future analyses using large sample sizes may improve the mapping of *trans*-acting genes.

TWAS uses the phenotype as the dependent variable and the predicted gene expression as an explanatory variable, while GSOR uses the predicted trait as an independent variable and uses the measured gene expression as a dependent variable. Both GSOR and TWAS are simply testing the null hypothesis that there are no genetic associations. Therefore, the interpretation of the results from GSOR is the same as TWAS: they both find a genetic association, between two traits, i.e. gene expression and GWAS trait, without implying causation. In TWAS, it is a genetic prediction of gene expression and phenotype of the trait. Since the individuals are different, the only covariance is genetic. In GSOR, the logic is the same. The *cis*-EBV or *cis*-PGS for the trait is based on SNPs, and the only covariance with expression in different individuals must be genetic. GSOR is determined by the accuracy of predicted phenotypes. TWAS is determined by the accuracy of predicted gene expression. We have shown that regardless of the use of GSOR or TWAS, traits with higher heritability, which are more genetically predictable, have more accurate outcomes in detecting associations (Figs. 1 and S1).

Applying GSOR and MTAO to the large dataset with transcriptomes and complex traits in cattle, we identified widespread genetic regulatory effects on complex traits, i.e. *cis*-pleiotropic effects, mediated by the transcriptome. We show that on average, each gene expression and splicing event mediates *cis*-genetic effects on 10 and eight traits, respectively, and this is comparable with our previous work where on average each variant affects 10 traits (33). This suggests that the genetic effects mediated by *cis*-regulatory mechanisms on complex traits are prevalent. Using a multitrait meta-analysis of SMR (16), a different approach to detect putative causal relationships between omics features and traits, we validate the results of MTAO. Also, we found that MTAO-prioritized genes show significantly stronger purifying selection than random genes, supporting that these genes have important functions in cattle and other mammals. These pieces of evidence support that MTAO can be used to identify omics features, i.e. regulatory elements, of high importance to complex traits. Apart from gene expression and splicing, there are many other types of quantitative omics features such as the height of ChIP-seq peaks (34) and allele-specific imbalance (25) which can also be analyzed by MTAO. Moreover, MTAO is summary data based; hence, it can be applied to any results from GSOR or conventional TWAS, as long as there are beta and standard error estimates for the association between the omics feature and the phenotype. Therefore, we expect MTAO to have an important place in future large-scale meta-analyses of different GSOR or TWAS studies in mammalian species.

We combined the results from the GSOR and SMR to prioritize tissues related to different phenotypes. The significant agreement between GSOR and SMR which is a single-*cis*-eQTL form for TWAS (16), also supports the robustness of our results. Across different traits, blood, milk cells, liver, and uterus were the most informative tissues for traits analyzed. Blood is one of the most common tissues/cell types sampled for omics studies due to its easy access. Milk cells can also be relatively easily accessed in dairy cattle (35). Although we have adjusted the analysis according to the sample size of each tissue, the tissue prioritization might still have some biases towards those with larger sample sizes.

Understanding the causal nature of large-effect QTL provides opportunities for treatments. Here, we used results from MTAO and SMR to dissect the regulatory effects of some major cattle loci, including *DGAT1*. A protein-coding mutation in *DGAT1* was previously identified as the cause of this QTL's effect on milk production traits, but there has been speculation that there are multiple causal variants in this region of the genome (12, 13). Our results, for the first time, show a correlation between *DGAT1* expression and splicing and numerous traits. *DGAT1* expression in blood, liver, mammary gland, pituitary, milk cells, and rumen was correlated with milk and nonmilk traits. This agrees with the pleiotropy model that we previously proposed (36) which is that QTL with a large effect on one trait is likely to have small effects on other uncorrelated traits. For example, previously we identified pleiotropic effects of *DGAT1* on stature, and the current studies linked such effects to blood, liver, mammary gland, and uterus (Fig. 6a). These findings allow us to hypothesize that the link between *DGAT1* and stature may be related to the biological pathways of milk production, fat metabolism, and musculoskeletal growth in early development. Future wet lab experiments to validate this hypothesis are needed. We did not observe any significant regulatory associations at *GHR* with cattle traits. Previously, protein-coding variants at *GHR* were found to be associated with cattle traits (24). Therefore, our work is in line with the evidence that QTL at GHR alters growth performances by changing protein coding rather than being regulatory.

In addition to *DGAT1*, the expression and splicing of four other genes (*ZNF34*, *IQANK1*, *LYNX1*, and *SPAG1*) close to *DGAT1* were correlated with complex traits. Similar results were observed for other loci like *MGST1*, *MUC1*, and *CSF2RB*. This could be because mutations could affect the regulation of more than one nearby gene, or it could be that these genes affect dairy traits directly. It has been demonstrated that multiple causal variants underlie human QTL (37). This may be the same for many traits of cattle, although we will need further experiments to validate this. One potential approach is for GSOR to include a fine mapping function similar to cTWAS (38) where predicted genetic components and SNPs are all fitted in one model. Such an extension to GSOR will be an important future goal.

We also provide statistical evidence for several new loci potentially affecting cattle traits via gene expression regulation, including blood group genes (*ABO* and *ACHE*). Interestingly, a deletion in *ABO* has been causally linked to pig complex traits via regulation of gut microbes (39) and here we found the regulatory effects of *ABO* in the jejunum (Table 1). In cattle, Tiplady et al. (40) showed a strong association between *ABO* gene expression and genetic signals for several milk fourier transform infrared (FTIR) wavenumbers, with the hypothesis that this was due to changes in milk oligosaccharide production or composition. However, there have not been reports regarding the effects of expression and splicing on complex traits linked to *ABO* or *ACHE* (Cartwright blood group). To our knowledge, it is the first study to find *DCXR* related to glucose metabolism and *FLII* related to prenatal development to have regulatory QTL in cattle.

Several improvements will be implemented in GSOR. First, currently, we use gBLUP to train the genomic predictor of phenotypes. It is known that BayesR provides more accurate predictions at the cost of high computing resources (41, 42). Therefore, the next step is to use the more efficient BayesR program, which can analyze tens of millions of variants in >100K animals, to construct predictive equations of traits. Second, while genomic prediction algorithms that jointly analyze variants are preferred in animals, the PRS approach leveraging variant effects from GWAS is popular in humans (3). Therefore, implementing PRS-based genomic predictors of phenotypes in GSOR will enhance its use in human data. Third, although we have enabled GSOR to take GRM and/or genetic PCs to minimize the bias from population structures, a systematic study of the impact of cross-population prediction of phenotypes on GSOR is required in the future. A study that quantifies the effect of population structure on the accuracy of prediction of phenotypes and omics data and how these will impact the analysis of GSOR and TWAS is needed in the future.

In conclusion, we have introduced new methods and meta-analysis strategies to link omics information and complex traits. The advantage of GSOR is the use of the *cis*-EBV or *cis*-PGS obtained from large datasets with genotypes and phenotypes. These methods are supported by the analysis of simulated and real data and by established Mendelian randomization methods which account for LD. Our methods detected widespread pleiotropic effects mediated by multiple regulatory mechanisms and prioritized many genes and splicing events with widespread phenotypic effects. Primarily developed in cattle, GSOR and summary-data-based MTAO can prioritize informative omics-phenotype associations in any species.

## Brief methods

Please see the full methods in the Supplementary material.

The RNA-seq data include new data from blood samples taken from 390 lactating cows from two breeds and milk samples from 281 lactating cows from two breeds. The other RNA-seq data from 15 tissues were obtained from the CattleGTEx website http://cgtex.roslin.ed.ac.uk/.

The genotype data for Australian animals, including those used for e/sQTL mapping and association analysis of phenotypes (described later), consisted of 16,251,453 sequence variants imputed using Run7 of the 1000 Bull Genomes Project (43, 44). The details of the imputation were described previously (20).

Phenotype data were collected by farmers and processed by DataGene Australia (http://www.datagene.com.au/) for the official May 2020 release of national breeding values. In total, 37 traits of 8,949 bulls and 103,350 cows, including Holstein (6,886♂/87,003♀), Jersey (1,562♂/13,353♀), cross-breed (36♂/5,037♀), and Australian Red (265♂/3,379♀) dairy breeds, were studied related to milk production, mastitis, fertility, temperament, and body conformation, and the details of these traits can be found in (20). No live animal experimentation was required.

A key feature of GSOR is the use of predicted phenotype value, i.e. genetic score (also called EBV or PGS (45)), from a large reference population, as the explanatory variable to be associated with gene expression levels, splicing events, or other omic features. Another key feature of GSOR was the use of variants close to the gene whose expression is being studied to calculate a local or *cis*-EBV/PGS. This would then be correlated with the expression or splicing of the gene. Note that although the local EBV/PGS was based on the effects of SNPs near the gene, all SNP effects are trained jointly (described below), where the total EBV/PGS minus the *cis*-EBV/PGS was the *trans*-EBV/PGS. It is generally recommended to use trait variant prediction models that jointly fit all variants together, such as gBLUP (46, 47) or BayesR (41, 48). Here, we considered gBLUP for computational efficiency. We also compared the analysis of GSOR with conventional TWAS, including mixed model-based method, and OSCA (49) and PrediXcan (2). To compare different methods, we performed extensive simulations including causal variants for gene expression and traits and also scenarios where variants causing gene expression also cause phenotypes. We combined results across traits to study omics-mediated *cis* pleiotropy and compared such results with SMR (50) and related both results to evolutionary constraints (d*N*/d*S*). We also used a heuristic index to explore the tissue relevance for traits. More details of GSOR are described in the “Method details” in the Supplementary material.

## Author list of the CattleGTEx consortium (v0)

Shuli Liu^#,1,2,3^, Yahui Gao^#,1,4^, Oriol Canela-Xandri^#,5^, Sheng Wang^#,6^, Ying Yu^#,2^, Wentao Cai^7^, Bingjie Li^8^, Ruidong Xiang^9,10^, Amanda J Chamberlain^10^, Erola Pairo-Castineira^5,11^, Kenton D'Mellow^5^, Konrad Rawlik^11^, Charley Xia^11^, Yuelin Yao^5^, Pau Navarro^5^, Dominique Rocha^12^, Xiujin Li^13^, Ze Yan^2^, Congjun Li^1^, Benjamin D Rosen^1^, Curtis P Van Tassell^1^, Paul M Vanraden^1^, Shengli Zhang^2^, Li Ma^4^, John B Cole^1^, George E Liu^14^, Albert Tenesa^15,16^, Lingzhao Fang^17,18,19^

^1^Animal Genomics and Improvement Laboratory, Henry A. Wallace Beltsville Agricultural Research Center, Agricultural Research Service, USDA, Beltsville, MD, USA.

^2^National Engineering Laboratory of Animal Breeding, College of Animal Science and Technology, China Agricultural University, Beijing, China.

^3^School of Life Sciences, Westlake University, Hangzhou, China.

^4^Department of Animal and Avian Sciences, University of Maryland, College Park, MD, USA.

^5^MRC Human Genetics Unit at the Institute of Genetics and Cancer, The University of Edinburgh, Edinburgh, UK.

^6^State Key Laboratory of Genetic Resources and Evolution, Kunming Institute of Zoology, Chinese Academy of Sciences, Kunming, China.

^7^Institute of Animal Science, Chinese Academy of Agricultural Science, Beijing, China.

^8^Scotland's Rural College (SRUC), Roslin Institute Building, Midlothian, UK.

^9^Faculty of Veterinary & Agricultural Science, The University of Melbourne, Parkville, Victoria, Australia.

^10^Agriculture Victoria, AgriBio, Centre for AgriBiosciences, Bundoora, Victoria, Australia.

^11^The Roslin Institute, Royal (Dick) School of Veterinary Studies, The University of Edinburgh, Midlothian, UK.

^12^INRAE, AgroParisTech, GABI, Université Paris-Saclay, Jouy-en-Josas, France.

^13^Guangdong Provincial Key Laboratory of Waterfowl Healthy Breeding, College of Animal Science & Technology, Zhongkai University of Agriculture and Engineering, Guangzhou, China.

^14^Animal Genomics and Improvement Laboratory, Henry A. Wallace Beltsville Agricultural Research Center, Agricultural Research Service, USDA, Beltsville, MD, USA. George.Liu@usda.gov.

^15^MRC Human Genetics Unit at the Institute of Genetics and Cancer, The University of Edinburgh, Edinburgh, UK. Albert.Tenesa@ed.ac.uk.

^16^The Roslin Institute, Royal (Dick) School of Veterinary Studies, The University of Edinburgh, Midlothian, UK. Albert.Tenesa@ed.ac.uk.

^17^Animal Genomics and Improvement Laboratory, Henry A. Wallace Beltsville Agricultural Research Center, Agricultural Research Service, USDA, Beltsville, MD, USA. lingzhao.fang@qgg.au.dk.

^18^MRC Human Genetics Unit at the Institute of Genetics and Cancer, The University of Edinburgh, Edinburgh, UK. lingzhao.fang@qgg.au.dk.

^19^Center for Quantitative Genetics and Genomics, Aarhus University, Aarhus, Denmark.

## Supplementary Material

pgaf208_Supplementary_Data

## Data Availability

The newly generated RNA-seq data (356 blood and 268 milk cells) will be made public via NCBI SRA (PRJNA392196, PRJNA616134, PRJNA305942, PRJNA392196, and PRJNA917329). Other RNA-seq data can be accessed via the CattleGTEx consortium (http://cgtex.roslin.ed.ac.uk/). Summary statistics for genes and splicing events associated with 37 traits of 110,000 cows across 16 tissues are publicly available at https://figshare.com/s/c10ffab5abf329b1318f. The Run7 imputation reference database (3,091 sequences) was developed by the 1000 Bull Genomes project members (51, 52). Full access to this database is available to members, and access can also be requested by external collaborators by emailing the following 1000 Bull Genomes project members: the corresponding author of this paper (ruidong.xiang@agriculture.vic.gov.au) and one of the steering committee members (amanda.chamberlain@agriculture.vic.gov.au). Additionally, a large portion of the 1000 Bull Genome Project imputation reference database has been publicly released (2,790 sequences), and these can be accessed via Run8 (https://www.ebi.ac.uk/ena/browser/view/ERZ1738264) and Run9 (https://www.ebi.ac.uk/ena/browser/view/PRJEB56689). DataGene Australia (http://www.datagene.com.au/) is the custodian of the raw phenotype and genotype data of Australian farm animals. Access to these data for research requires permission from DataGene under a Data Use Agreement. Other supporting data are shown in the Supplementary Materials of the manuscript. Code and tutorials for GSOR and MTAO are available at https://github.com/rxiangr/GSOR-and-MTAO. The linear mixed model analysis used GCTA (53).

## Preprints

This manuscript was posted on a preprint at https://doi.org/10.1101/2022.07.13.499886.
