# Supplementary material for: Genetic score omics regression and multitrait meta-analysis detect widespread cis-regulatory effects shaping bovine complex traits
Source: PNAS Nexus. 2025 Jul 2;4(7):pgaf208. doi: 10.1093/pnasnexus/pgaf208 (PMC12275098; doi:10.1093/pnasnexus/pgaf208)
Supplement: pgaf208_Supplementary_Data [file pgaf208_supplementary_data.zip › PNASNEXUS-PNASNEXUS-2024-00738-TRR-s01.docx]

**Supplementary information for:**

**Genetic score omics regression and multi-trait meta-analysis detect widespread *cis*-regulatory effects shaping bovine complex traits**

Ruidong Xiang^1,2,*^, Lingzhao Fang^3^, Shuli Liu^4^, George E. Liu^5^, Albert Tenesa^3,6^, Yahui Gao^5^, Brett A Mason^1^, Amanda J. Chamberlain^1^, Michael E. Goddard^1,2^

^1^ Agriculture Victoria, AgriBio, Centre for AgriBiosciences, Bundoora, VIC, 3083, Australia

^2^ School of Agriculture, Food and Ecosystem Sciences, The University of Melbourne, Parkville, VIC, 3052, Australia

^3^ MRC Human Genetics Unit at the Institute of Genetics and Cancer, The University of Edinburgh, Edinburgh, United Kingdom

^4^ Westlake Laboratory of Life Sciences and Biomedicine, Hangzhou, Zhejiang, 310024, China

^5^ Animal Genomics and Improvement Laboratory, Henry A. Wallace Beltsville Agricultural Research Center, Agricultural Research Service, Agricultural Research Service, USDA, Beltsville, Maryland 20705, USA

^6^ The Roslin Institute, Royal (Dick) School of Veterinary Studies, The University of Edinburgh, Midlothian EH25 9RG, UK

This file contains:

Method details.

Supplementary Note 1.

Supplementary Figures 1-10.

Supplementary Tables 1-9.

Full author list of the CattleGTEx consortium (v0)

**Method details**

**RNA-seq data**. The RNA-seq and genotype data analysed included those generated by Agriculture Victoria Research (AVR) in Victoria, Australia, and those provided by the CattleGTEx consortium ^14^ (Supplementary Table 2). The animal ethics was approved by the DJPR Animal Ethics Committee (application numbers 2013-14 and 2018-2019), Australia. Blood samples were taken from 390 lactating cows from 2 breeds, and milk samples from 281 lactating cows from 2 breeds. The processing of samples, RNA extractions, and library preparation followed that previously described ^54,55^. RNA sequencing (RNA-seq) was performed on a HiSeq3000 (Illumina Inc) or NovaSeq6000 (Illumina Inc) genome analyzer in a paired-end, 150-cycle run. Only RNA-seq data of 356 Holstein and 26 Jersey with > 50 million reads for milk cells or > 25 million reads for white blood cells and had concordant alignment rate ^56^ > 80% were used. QualityTrim (https://bitbucket.org/arobinson/qualitytrim) was used to trim and filter poor-quality bases and sequence reads. Adaptor sequences and bases with a quality score of <20 were removed. Reads with a mean quality score less than 20, greater than 3 N, greater than three consecutive bases with a quality score less than 15, or a final length of fewer than 50 bases were discarded. High-quality raw reads were aligned to the ARS-UCD1.2 bovine genome ^57^ with STAR ^56^ using the 2-pass method. The gene counts were extracted by FeatureCount ^58^. Leafcutter ^30^ was used to generate junction files which were then used to create the RNA splicing phenotype matrix, i.e., intron excision ratio ^30^.

The RNA-seq gene counts of 15 tissues (Supplementary Table 2) where the sample size > 100 were downloaded from CattleGTEx website <http://cgtex.roslin.ed.ac.uk/>. The blood counts generated by AVR (white blood cells) and CattleGTEx were combined. All gene counts were normalised by voom ^59^ and then underwent quantile normalisation for the following analyses. Junction files from CattleGTEx tissues were also downloaded and data from each tissue was processed with leafcutter ^30^ to generate RNA splicing phenotype. Milk cell data used in this study was only from AVR.

**Genotype data**. The genotype data for Australian animals including those used for e/sQTL mapping (blood and milk cells) and association analysis of phenotypes (described later) consisted of 16,251,453 sequence variants imputed using Run7 of the 1000 Bull Genomes Project ^44,45^. The details of the imputation were described previously ^46^. Briefly, the imputation of bi-allelic sequence variants was performed with Minimac3 ^60,61^ and those variants with imputation accuracy *R^2^* > 0.4 and minor allele frequency (MAF) > 0.005 in both bulls and cows were kept. Bulls were genotyped with either a medium-density SNP array (50K: BovineSNP50 Beadchip, Illumina Inc) or a high-density SNP array (HD: BovineHD BeadChip, Illumina Inc) and cows were genotyped with the BovineSNP50 Beadchip (Illumina Inc). The genotype data for CattleGTEx animals were generated previously ^14^ and included a total of more than 6 million sequence variants imputed also using Run7 of the 1000 Bull Genomes Project. Those variants with the imputation dosage R-squared > 0.8 and MAF > 0.001 were kept.

**Phenotype data**. Data were collected by farmers and processed by DataGene Australia (<http://www.datagene.com.au/>) for the official May 2020 release of National breeding values. No live animal experimentation was required. DataGene provided the bull and cow phenotypes as de-regressed breeding values or trait deviations for cows, and daughter trait deviations for bulls (i.e., progeny test data for bulls). DataGene corrected the phenotypes for herd, year, season, and lactation following the procedures used for routine genetic evaluations in Australian dairy cattle. Phenotype data included a total of 8,949 bulls and 103,350 cows, including Holstein (6,886♂ / 87,003♀), Jersey (1562♂ / 13,353♀), cross-breed (36♂ / 5,037♀) and Australian Red (265♂ / 3,379♀) dairy breeds. In total, 37 traits were studied that related to milk production, mastitis, fertility, temperament, and body conformation and the details of these traits can be found in ^46^. For AVR blood samples, breed and days in milk (DIM) were fitted as fixed effects in the gene expression and splicing GWAS model. For the milk samples, experiment, DIM, and the first and second principal components, extracted from the expression count matrix, were fitted as fixed effects. Principal components were fitted to adjust for the high expression of the major milk protein genes, i.e., casein, in milk cells based on previous experiences ^54^.

**Genetic Score Omics Regression (GSOR)**. A key feature of GSOR is the use of predicted phenotype value, i.e, genetic score (also called estimated breeding value or polygenic score), from a large reference population, as the explanatory variable to be associated with gene expression levels, splicing events, or other omic features. Another key feature of GSOR was the use of variants close to the gene whose expression is being studied to calculate a local or *cis* EBV/PGS. This would then be correlated with the expression or splicing of the gene. Note that although the local EBV/PGS was based on effects of SNPs near the gene, all SNP effects are trained jointly (described below). Where the total EBV/PGS minus the cis EBV/PGS was the trans EBV/PGS. It is generally recommended to use trait variant prediction models that jointly fit all variants together, such as gBLUP ^47,48^ or BayesR ^42,49^. Here we considered gBLUP for computational efficiency. A basic gBLUP model can be described as:

$y_{P}=X\beta+ZWu+e$ (4)

or

$y_{P}=X\beta+Zg+e$ (5)

Where is $X$ a design matrix, $y_{P}$ is an n × 1 vector of phenotypes and n is the number of individuals; $\beta$ is a vector of fixed effects and Z is the matrix allocating records to individuals (place holder for individual animal ID); u is a vector of SNP effects with u ~ N(0, $I\sigma_{u}^{2}$) where I is an n × n identity matrix; $W$ is a standardized genotype matrix and if models like GCTA ^53^ were used, $W_{ij}=\frac{(x_{ij}-2p_{i})}{\sqrt{2p_{i}(1-p_{i})}}$ where $x_{ij}$ is the number of copies of the 1^st^ allele for the i^th^ SNP of the j^th^ individual and $p_{i}$ is the frequency of the 1^st^ allele; $g$ is an n × 1 vector of the total genetic effects of the individuals with g ~ N(0, $G\sigma_{g}^{2}$) where $G$ is the genomic relationship matrix (GRM) between individuals, $G=WW'\sigma_{u}^{2}/\sigma_{g}^{2}$ or$G=\frac{WW'}{M}$ where $\sigma_{g}^{2}=M\sigma_{u}^{2}$ and M is the number of variants to explain $\sigma_{g}^{2}$; and e is the residual where e ~ N(0,$I\sigma_{e}^{2}$). As Equation (4) and (5) are equivalent ^48,53,62^, it is possible to transform the BLUP of individual genetic score $g$ to BLUP of $\hat{u}$, i.e., SNP effects jointly estimated:

$\hat{u}=\frac{W^{'}G^{-1}g}{M}$ (6)

Equation 6 was implemented with GCTA BLUP ^53^ in the current study. Estimated $\hat{u}$ can be used to predict the genetic score, i.e., breeding value or polygenic score, of new individuals based on their genome-wide variant data: $\hat{g}=W_{new}\hat{u}$. Because the SNP effects $\hat{u}$ was jointly estimated, it is also possible to use a subset of variants to predict $\hat{g}$ (local EBV/PGS). For example, we have previously estimated $\hat{g}$ of every 50kb windows of variants ^63^. In the current study, we estimate $\hat{g}$ using variants close or distant to omic features such as genes or introns:

$$\left\{ \begin{aligned} \hat{g}_{P_{cis}}=W_{cis}\hat{u}_{cis} (7) \\ \hat{g}_{P_{trans}}=W_{trans}\hat{u}_{trans} (8) \\ \hat{g}_{P_{total}}=\hat{g}_{P_{cis}}+\hat{g}_{P_{trans}} (9) \end{aligned} \right.$$

Where $\hat{g}_{P_{cis}}$ is the estimated genetic score using effects on phenotype ($\hat{u}_{cis}$) and the genotype matrix ($W_{cis}$) of *cis* variants of omic features; $\hat{g}_{P_{trans}}$ is the estimated genetic score using effects on phenotype ($\hat{u}_{trans}$) and the genotype matrix ($W_{trans}$) of *trans* variants; and $\hat{g}_{P_{total}}$ is the total genetic score by summing the *cis* and *trans* estimations. In the GSOR, for each gene, the *cis* variants were defined as ±1Mb of the transcription start site of the gene and the *trans* variants are the remaining variants. For each intron, *cis* variants were defined as those within 1Mb down and upstream of the intron (from intron start – 1Mb to intron end + 1Mb) and the *trans* variants are the remaining variants. Once *cis* and/or *trans* estimated genetic scores of genes/introns were obtained, they were analysed as response variables with gene expression or RNA splicing (intron excision ratio) as predictors:

$$\left\{ \begin{aligned} \hat{g}_{P_{cis}}=\Omega b_{cis}+\mathbf{X}b_{\boldsymbol{\Omega}}+{\boldsymbol{(}\boldsymbol{a}}_{\boldsymbol{\Omega}})+e (10) \\ \hat{g}_{P_{trans}}=\Omega b_{trans}+\mathbf{X}b_{\boldsymbol{\Omega}}+{\boldsymbol{(}\boldsymbol{a}}_{\boldsymbol{\Omega}})+e (11) \\ \hat{g}_{P_{total}}=\Omega b_{total}+\mathbf{X}b_{\boldsymbol{\Omega}}+(\boldsymbol{a}_{\boldsymbol{\Omega}})+e (12) \end{aligned} \right.$$

Where $\Omega$ is an n × 1 vector of omics values such as gene expression or RNA splicing corrected for other fixed effects such as breed, sex and experiments, $b_{cis}$ is the regression coefficient of the *cis* estimated genetic score $\hat{g}_{cis}$ $on \Omega$, $b_{trans}$ is the regression coefficient of the trans estimated genetic score $\hat{g}_{trans}$ on $\Omega$, and $b_{total}$ is the regression coefficient of the total genetic score $\hat{g}_{total}$ on $\Omega$; $\mathbf{X}$ was the design matrix for fixed effects for data with omics measurements, e.g., breeds; $b_{\boldsymbol{\Omega}}$ was the vector of fixed effects in the omics data; $\boldsymbol{a}_{\boldsymbol{\Omega}}$ was a vector of random polygenic effects ~N(0, **G**σ_g_^2^) which can be optionally fit to adjust confounding factors, **G** = genomic relatedness matrix (GRM) based on all variants and σ_g_^2^ = random polygenic variance, and e is the residual.

The implementation of GSOR was undertaken in R (v4.0.0) and is publicly available at (<https://github.com/rxiangr/GSOR-and-MTAO>). GSOR can work with or without random effects and when it does, it uses the implementation of eigendecomposition of the relationship matrix to speed up the variance components analysis. In the AVR high-performance cluster (slurm) system with 1 node, GSOR used 1.6G RAM and took 4.5 minutes to associate expression levels of 16,564 genes with the trait genetic score of 945 individuals fitting a GRM for each regression.

**Conventional Transcriptome-Wide Association Studies (TWAS)**. Opposite to GSOR, a conventional TWAS essentially associates predicted gene expression in a large population with phenotypes of this population. The variant predictor was directly trained in the population where omics data was available. To make results from GSOR and TWAS comparable, we conducted TWAS using linear mixed model approaches and the variant predictors were trained using the omics data from blood which had the largest sample size across all tissues analysed. To train the variant predictor, a 2-GRM model was analysed for each omic feature which is similar to equation 5:

$y_{\Omega}=X\beta+Z\hat{g}_{\Omega_{cis}}+Z\hat{g}_{\Omega_{trans}}+e$ (13)

Where $y_{\Omega}$ is an n × 1 vector of omics values such as gene expression or RNA splicing, $\beta$ is a vector of fixed effects; $g_{\Omega_{cis}}$ is an n × 1 vector of the total genetic effects of the individuals with g ~ N(0, $G_{cis}\sigma_{g}^{2}$) where $G_{cis}$ is the GRM built by *cis* variants of the omic feature; $g_{\Omega_{trans}}$ is an n × 1 vector of the total genetic effects of the individuals with g ~ N(0, $G_{trans}\sigma_{g}^{2}$) where $G_{trans}$ is the GRM built by *trans* variants of the omic feature; Z is the matrix allocating records to individuals; e is the error term. Once $\hat{g}_{\Omega_{cis}}$ and $\hat{g}_{\Omega_{trans}}$ were obtained, equation (6) was applied to estimate SNP BLUP for omics data: $\hat{u}_{\Omega_{cis}}$, $\hat{u}_{\Omega_{trans}}$ and $\hat{u}_{\Omega_{total}}$, which were used to predict the omics scores, $\hat{g}_{\Omega_{cis}}$, $\hat{g}_{\Omega_{trans}}$ and $\hat{g}_{\Omega_{total}}$ in the population with phenotypic records with equations 7-9. Then, predicted gene expression values were analysed as explanatory variables to associate with phenotypes:

$$y_{P}=\left\{ \begin{aligned} \hat{g}_{\Omega_{cis}}\beta_{cis}+\mathbf{X}b_{P}+(\boldsymbol{a}_{P})+ e (14) \\ \hat{g}_{\Omega_{trans}}\beta_{trans}+\mathbf{X}b_{P}+(\boldsymbol{a}_{\mathbf{P}})+e (15) \\ \hat{g}_{\Omega_{total}}\beta_{total}+\mathbf{X}b_{P}+(\boldsymbol{a}_{P})+e (16) \end{aligned} \right.$$

Where $y_{P}$ is an n × 1 vector of phenotypes, $\beta_{cis}$ is the regression coefficient for *cis* estimated omics score $\hat{g}_{\Omega_{cis}}$, $\beta_{trans}$ is the regression coefficient for *trans* estimated omics score $\hat{g}_{\Omega_{trans}}$, and $\beta_{total}$ is the regression coefficient for the total omics score $\hat{g}_{total}$; $\mathbf{X}$ was the design matrix for fixed effects, e.g., breeds; $b_{P}$ was the vector of fixed effects in the dataset with phenotypic records; $\boldsymbol{a}_{P}$ is random effects based on the genomic relationships between individuals with phenotypic data which can be optionally fit to adjust confounding factors and e is the residual. The training of variant predictors of omics data used gBLUP implementation of MTG2 and the TWAS used the implementation of OSCA ^50^. In addition, we also implemented TWAS using the elastic net model implemented in the PrediXcan ^2^. The files of genotype, gene expression, and annotation of SNPs and genes were prepared according to the instructions provided by <https://github.com/hakyimlab/PrediXcan>. Model parameters used the default options provided by the authors.

**Simulations**. To compare GSOR with TWAS, we simulated data where causal variants that affect gene expression and phenotypes were overlapped. We used the 6 million real sequence genotypes from the blood dataset to simulate 16,600 gene expression phenotypes with the following framework: 1) gene coordinates from bovine ARS-UCD1.2 reference genome were used; 2) the expression of each gene had 1-2 causal cis eQTL and 0-3 causal trans eQTL (on different chromosome to the gene) so that all genes had causal cis eQTL but not all genes had causal trans eQTLand genes had causal cis eQTL only or both causal cis and trans eQTL; 3) across 16,600 genes, 1049 had causal cis and/or trans eQTLs overlapping with causal QTL under the alternative scenario (described later) 268 of which had causal trans eQTL overlapping with causal QTL; 4) in total, 1,771 causal eQTL in the expression data were also causal QTL; 5) the effects of cis causal eQTL were randomly sampled from a uniform distribution where the minimum was 0.05 and the maximum was 0.5; 6) the effects of trans causal eQTL were randomly sampled from a uniform distribution where the minimum was 1e-6 and maximum was 0.05; this was to make average effects of trans eQTL 10 times smaller than cis eQTL; 7) the heritability of the expression of genes was sampled from a normal distribution where the mean *h*^2^ was 0.25 and the standard deviation was 0.2; only positive values were allowed.

Sixteen million sequence genotypes from more than 100K cows were used to simulate cow phenotypes with the following framework: 1) 5000 causal variants were defined and used to simulate 10 traits; 2) the first 5 traits (1-5) were simulated under the null where their 5000 causal variants did not overlap with causal eQTLs. These 5 traits had heritabilities of 0.4, 0.5, 0.6, 0.65 and 0.7, respectively; 3) the second set of 5 traits (6-10) were simulated under the alternative scenario, with the same heritability settings, but the 5000 causal SNPs overlapped with causal eQTL as described above. All simulations used the framework from GCTA GWAS model ^53^.

To understand if the causal direction can affect the results, we performed additional simulations where we used above-simulated gene expression levels that contained causal variants to simulate phenotypes. This was to make sure that SNPs cause gene expression which causes phenotypes. We used OSCA ^50^ using expression of randomly selected 100 genes to simulate 5 traits with heritability of 0.1, 0.3, 0.5, 0.7 and 0.9. We then repeated the analyses of GSOR and TWAS described above to analyse newly simulated data.

**Comparison between GSOR and TWAS**. In simulations, a gene was defined as a causal gene if it had both causal eQTL and QTL, i.e, the same SNP was both eQTL and QTL. All genes analysed were then classified as causal or non-causal and this was analysed using the Receiver Operating Characteristic (ROC) curves against p-values from GSOR and TWAS. ROC analysis used the R package ‘pROC’ and resultant ROC curves were presented using ggplot2. In analysing real data, we compared gene-trait associations between cis and trans-predictions. In GSOR, both cis and trans variants were used to predict genetic scores to be associated with gene expression. In TWAS, both cis and trans variants were also used to predict omics values to be associated with phenotypes. For a gene, while we could not expect its association with a trait to be significant based on both cis and trans predictions, we could expect its trait association to have the same direction of effect in both cis and trans predictions. Therefore, we compared the proportion of significant genes with the same direction of effect in both cis and trans predictions between GSOR and TWAS. Genes with p < 0.05 in both cis ($\hat{g}_{\Omega_{cis}}$) and trans predicted ($\hat{g}_{\Omega_{trans}}$) analysis in GSOR or TWAS were used for the comparison. To test the statistical significance of ROC analyses, we used a t-test to compare the area under the curve calculated between null and alternative simulations and between GSOR and TWAS across multiple scenarios.

**Omics mediated cis pleiotropy**. GSOR estimates the effects (beta) and standard error of each gene expression or splicing event on a trait. Combining these results across traits could provide insights into pleiotropy. Focusing on the cis-predicted genetic score, we performed a meta-analysis to quantify the extent of multi-trait effects of each gene expression or splicing event. The results of the analysis indicated the extent of cis pleiotropy mediated by omics. For each gene expression or splicing event, the t value (beta/SE) from GSOR for each associated trait was obtained to model the number of traits ($N_{pleio}$) affected and the magnitude ($M_{pleio}$) of such pleiotropic effects. To estimate $N_{pleio}$, the t values across traits were decorrelated using the Mahalanobis transformation described by Jordan et al. 2019 ^19^. Then, we adopted the method from Jordan et al 2019 ^19^ with a more stringent significance test:

$N_{pleio}=n\left( \left| t_{i} \right|>2 \right)$ (17)

Where $n\left( \left| t_{i} \right|>2 \right)$ is the number of t values, out of the total number of K traits, of the omic feature *i* with a magnitude greater than 2. Two is used because it represents a standard t value in a normal distribution with a significance cutoff of p = 0.045. The significance test of $N_{pleio}$ used ${Prop}_{pleio}=\frac{(N_{pleio}-1)}{K}$ where ${Prop}_{pleio}$ is the proportion of traits significantly affected by the omics feature. To obtain the p-value, ${Prop}_{pleio}$ was then tested against the probability of 0.045 which is the probability of the t value being greater than 2 in the normal distribution. The reason for using $\frac{(N_{pleio}-1)}{K}$ instead of using $\frac{N_{pleio}}{K}$ (used by Jordan et al. 2019^19^) is when $N_{pleio}$= 1, i.e., the omics feature only affects one trait, then this does not qualify as pleiotropy, which is defined as genetic effects on more than 1 trait.

To estimate the magnitude of mediated pleiotropy, we used:

$M_{pleio}=\sqrt{{t_{i}}^{'}V^{-1}t_{i}}$ (18)

Where $t_{i}$ is the effects (beta/SE) from GSOR for each omics feature, ${t_{i}}^{'}$ is the transpose of $t_{i}$ and $V^{-1}$, $V$ is the K ×K correlation matrix based on the t values for each trait. To obtain the significance of $M_{pleio}$,${\sqrt{{t_{i}}^{'}V^{-1}t_{i}}}^{2}= {t_{i}}^{'}V^{-1}t_{i}$ was tested against the $\chi^{2}$ distribution with degrees of freedom K. This approach was adopted from Bolormaa 2014 et al. ^6^ and Xiang et al. 2017 and 2020 ^38,64^ and gives identical results using the method from Jordan et al 2019^19^, but without the need for decorrelation of t values. The Rscript to conduct the meta-analysis of $N_{pleio}$ and $M_{pleio}$ are publicly available at <https://figshare.com/s/c10ffab5abf329b1318f>.

**Summary data-based Mendelian Randomization (SMR)**. To verify the results from MTAO, we conducted SMR using mapped cis eQTL and sQTL (±1Mb from the gene or intron) from 16 tissues and GWAS results from 37 traits ^20^. Because cis eQTL or sQTL rely on SNPs very close to each other which usually have high LD, the heterogeneity in dependent instruments (HEIDI) ^16^ test is an effective analysis to distinguish causal from LD. The mapping of eQTL and sQTL are detailed in ^21^. Briefly, we first used a linear mixed model approach to map cis eQTL and sQTL in GCTA: $y_{\Omega}=X\beta+Zg_{all}+Wv+e$ (19); where $y_{\Omega}$ is an n × 1 vector of omics values such as gene expression or RNA splicing, $\beta$ is a vector of fixed effects like breeds, different experiments or PEER ^65^ factors; $g_{\Omega_{all}}$ is an n × 1 vector of the total genetic effects of the individuals with g ~ N(0, $G_{all}\sigma_{g}^{2}$) where $G_{all}$ is the GRM built by all the variants; $W$ is the design matrix of variant genotypes (0, 1, 2) and $v$ is the variant additive effect; e is the error term. We then saved the eQTL mapping results in the BESD format (<https://yanglab.westlake.edu.cn/software/smr/#BESDformat>), which is the required data format for SMR. We selected eQTL or sQTL with p < 5e-6 for SMR analysis and a multi-SNP-based SMR test was chosen. Because the RNA-seq data was based on worldwide cattle breeds, we used the 1000-bull whole-genome sequence run7 data ^51^ as the reference panel for SMR. As additional verification, we also applied MR-PRESSOR ^66^ to test the association using significant *cis* eQTLs and the GWASs of traits. Default settings of MR-PRESSOR were used and the number of iterations was up to 50,000. The p-values corrected for pleiotropic outliers were reported.

**Multi-trait meta-analysis of SMR and comparison with MTAO**. For a gene or an intron, SMR estimates beta and standard error for a trait, based on the top e/sQTL. Therefore, we can apply equation 21 to $t_{SMR}=\frac{b_{SMR}}{{se}_{SMR}}$ (20) obtained for different traits to test the hypothesis that a gene or an intron has effects on more than 1 trait. This meta-analysis also matched the framework of MTAO described above. After calculating the chi-square p-value of multi-trait SMR for each gene and intron similar to equation 18, in each tissue, we count the four following numbers: 1) total number of genes or introns testable between MTAO and SMR; 2) number of genes or introns with p < 0.05 in MTAO; 3) the number of genes or introns with p < 0.05 in multi-trait SMR and 4) the number of genes or introns with p < 0.05 in both MTAO and multi-trait SMR. Then, for each tissue, we used these four counts to generate a contingency table for a Fisher’s exact test [fisher.test(…, alternative='greater') in R v4.0.0] of the significance of the overlap between MTAO and SMR more than expected by random chance. The odds ratio of overlap was obtained from each fisher’s exact test and the p-value was adjusted for multi-testing. Additionally, genes identified by GSOR and SMR were analysed for GO term enrichment using pathfindR ^67^ using its default options. Then the number of significantly enriched pathways (FDR adjusted p < 0.05) was compared between GSOR and SMR.

**dN/dS**. We retrieved the dN and dS values precalculated by Ensembl (version 99) using R library biomaRt(). dN and dS values were retrieved between cattle (Bos taurus) and humans (Ensembl short label: hsapiens), between cattle and mouse (mmusculus), between cattle and pigs (sscrofa), between cattle and sheep (oaries), between cattle and goat (chircus) and between Bos taurus and Bos indicus (bihybrid, i.e., UOA_Brahman_1). Then the ratio was calculated as dN/dS for all genes participating in the analysis. Only genes with the orthology type as 1-to-1 homology were used in the analysis. The significance of the difference in means of log_2_(dN/dS) between all genes and MTAO prioritised genes was tested in a t-test.

**Relevant tissues for traits**. For results from GSOR for each tissue and trait, there is a beta and SE, and therefore, $t_{GSOR}$, estimated for each gene or splicing event. Also, there are results from SMR described above for each gene/splicing which can be combined with results from GSOR to prioritise informative tissues. The squared t-value of a SNP from GWAS can be used to estimate the amount of phenotypic variance explained ^20^. In the current study, to link different tissues to traits, we calculated the following heuristic index: $\frac{\sum_{1}^{N_{gene}} {[ ( t_{GSOR}\times\frac{t_{SMR}}{t_{HEIDI}})}^{2}-1]}{{log}_{2}[{(N_{indiv})}^{2}\times{(N_{gene})}^{2}]}$ (21) where the magnitude of effects of each gene or intron ($|t_{GSOR}|$) was adjusted by the magnitude of effect of the SMR test ($|t_{SMR}|$) and the HEIDI test (|$t_{HEIDI}|$), so that it is positively related to the effects from SMR and negatively related to the LD confounding from HEIDI. The sum of squares was also adjusted for the number of genes and individuals analysed for each tissue and the log scale adjustment made the denominator a linear variable like the numerator. Equation 21 was then used to prioritise informative tissues. Genes and introns were excluded from the analysis if their nominal p-value was > 0.05 in GSOR and SMR and p-value <0.05 in HEIDI test.


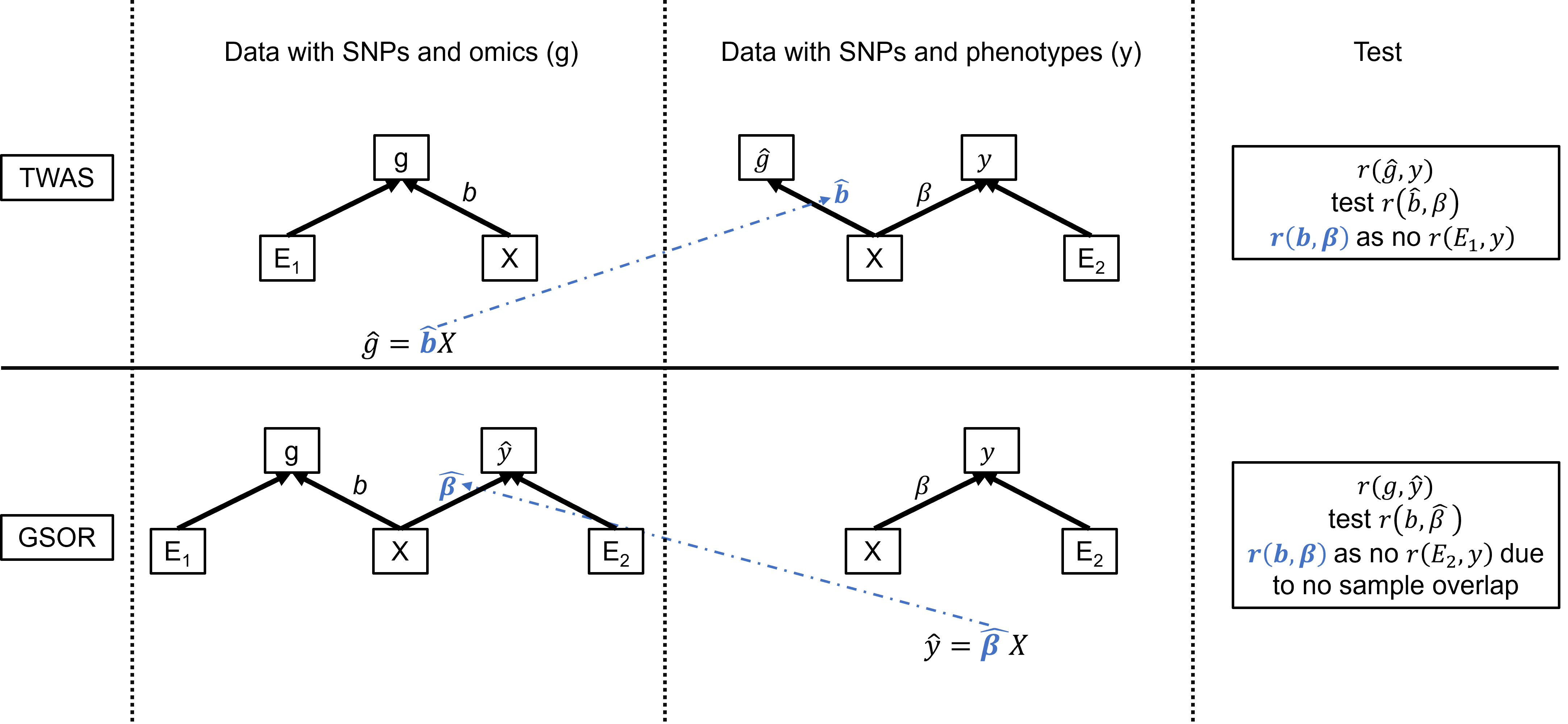


**Supplementary Note 1**. Illustration of statistical equivalence of statistical test between TWAS and GSOR.

In the upper left panel, TWAS first trains genetic models for omics data (e.g., gene expression or g) to estimate effects ($\hat{b}$) of genetic information (X), and then use $\hat{b}$ and genetic data (X) to predict omics data ($\hat{g}$) in the data where there are data of phenotypes (y, upper middle panel). In the dataset where there are phenotypes (upper middle panel), TWAS tests the correlation between $\hat{g}$ and y, i.e., $r(\hat{g},y$). Because genetic information (X) is the same in constructing $\hat{g}$ and $y$ , $r(\hat{g},y$) equals to testing the correlation between $\hat{b}$ and the real effects ($\beta$) of genetic information on y, i.e., $r\left( \hat{b},\beta\right)$. As environment effects in two datasets (E_1_ and E_2_) are independent, there is no need for testing $r\left( E_{1},y \right)$. In the null hypothesis that $r\left( b,\beta\right)$= 0 then $r\left( \hat{b},\beta\right)$=0 because there can be no environmental correlation.

In the lower middle panel, GSOR first trains genetic models for phenotypes (y) to estimate effects ($\hat{\beta}$) of genetic information (X), and then use $\hat{\beta}$ and genetic data (X) to predict y ($\hat{y}$). In the dataset where there are omics data (e.g., gene expression or g, lower left panel), GSOR tests the correlation between $r(g,\hat{y}$). Because genetic information (X) is the same in constructing $g$ and $\hat{y}$, $r(g,\hat{y}$) equals to testing the correlation between $\hat{\beta}$ and the real effects of genetic inforamtion ($b$) on g, i.e., $r\left( b,\hat{\beta} \right)$. As environment effects in two datasets (E_1_ and E_2_) are independent, i.e., no $r\left( E_{2},y \right)$. As there is no sample overlap, in the null hypothesis that $r\left( b,\beta\right)$=0 then $r\left( b,\hat{\beta} \right)$=0. Therefore, in both TWAS and GSOR, the effective test was the same, i.e., $r\left( b,\beta\right).$


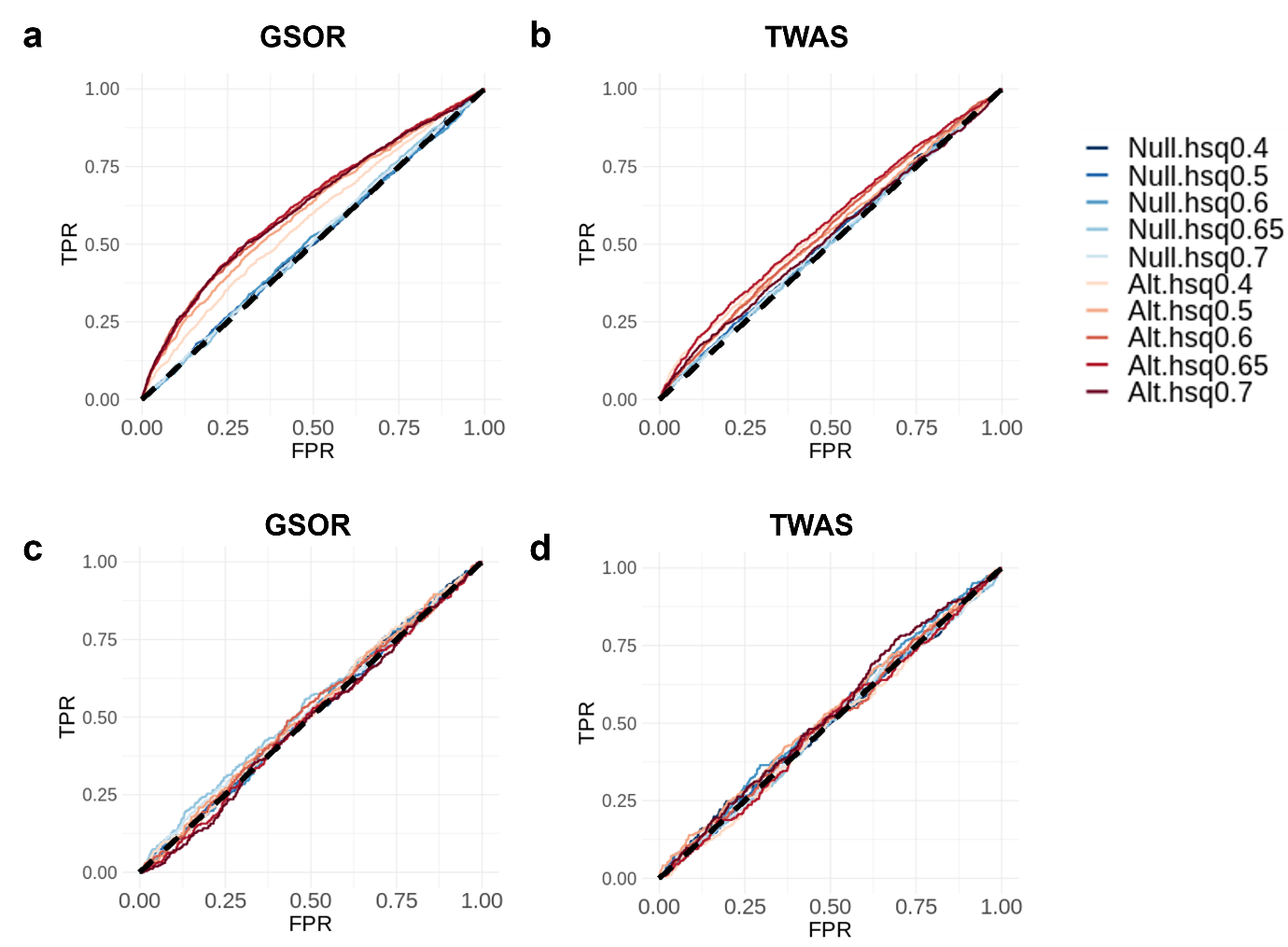


**Supplementary Figure 1**. Comparison of results between GSOR and TWAS using simulated data. Receiver Operating Characteristic (ROC) analysis of results from GSOR and TWAS based on cis+trans predicted values are shown in (**a**) and (**b**), respectively. ROC analysis of results based on trans predicted values are shown in (**c**) and (**d**). 10 scenarios were simulated with varying heritability (hsq) of traits. 5 traits were simulated under the null (Null) where no causal eQTL overlapped with causal QTL and another 5 traits were simulated under the alternative (Alt) scenarios where causal eQTL overlapped with causal QTL for more than 1000 genes.

**Supplementary Figure 2**. Agreement of the direction of association between cis and trans predicted values in the simulated data. The Y-axis indicates the number of simulated causal genes with an association p-value < 0.05 in both cis and trans analyses. N.diff.dir: the number of genes that had different direction of associations. N.same.dir: the number of genes that had the same direction of associations. The percentages on top of the bars indicate the proportion of genes showing the same direction of association between cis and trans predicted values.


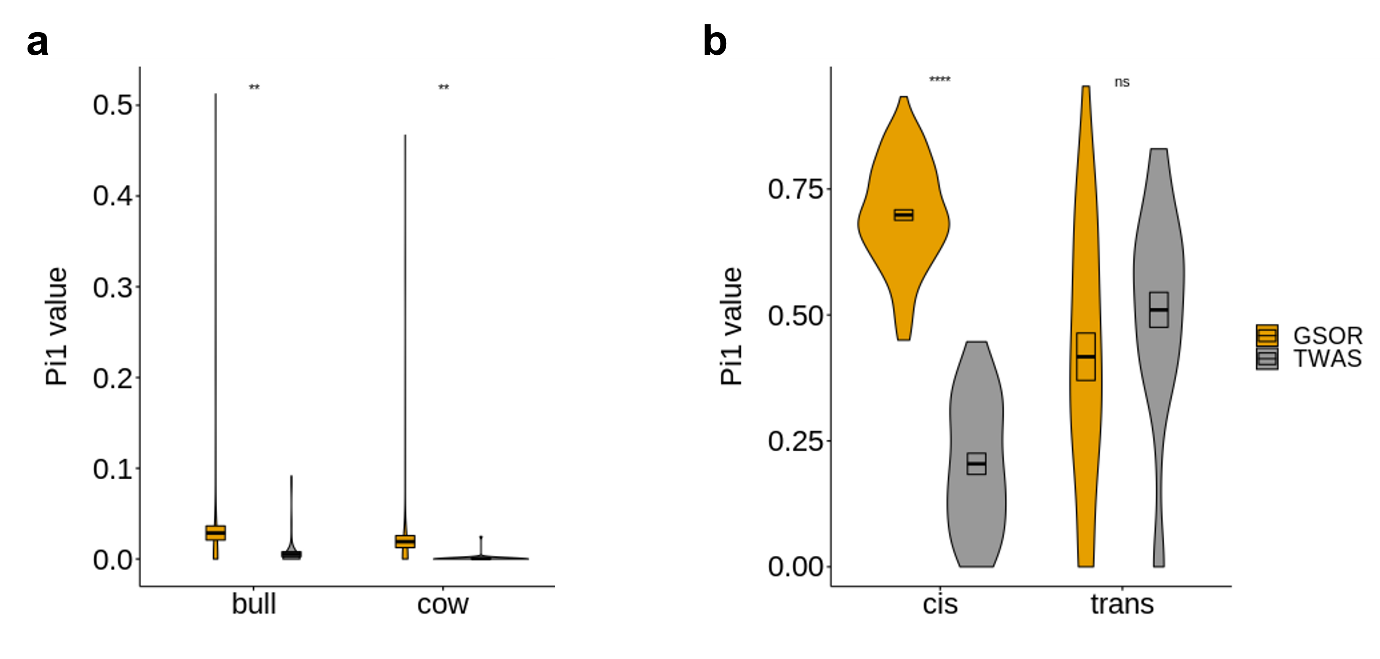


**Supplementary Figure 3**. Comparison of results between GSOR and TWAS using real data. **a**: $\pi_{1}$ value, an indication of the amount of replicated associations, of GSOR and TWAS across 37 traits between analyses using cis predicted and trans predicted values. Such replication was done in bulls and cows. **b**: $\pi_{1}$ value of GSOR and TWAS between the two sexes based on cis and trans predicted values. **: p < 0.01, ****: p < 0.0001 and ns: not significant.


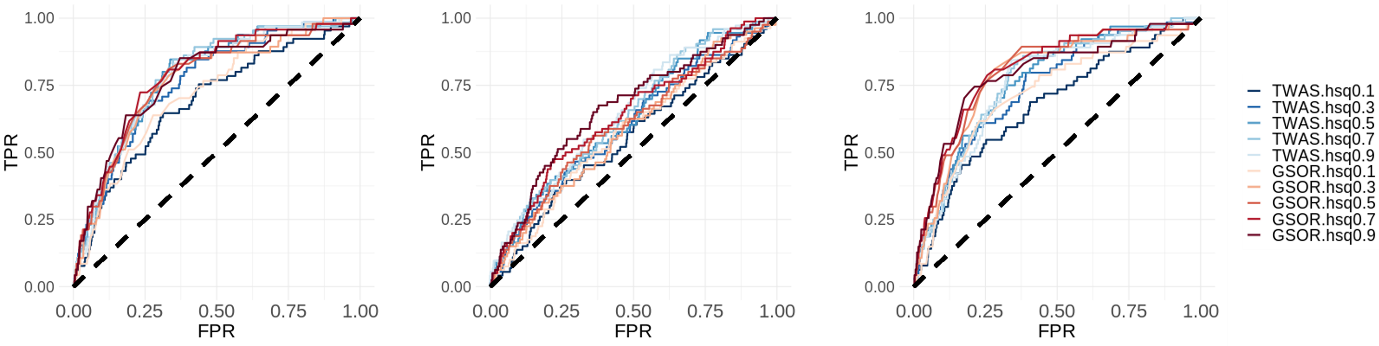


**Supplementary Figure 4**. Comparison of GSOR and TWAS using simulated data where 100 genes with causal SNPs were simulated to cause quantitative traits. Three panels from the left to right represent results from cis-predicted, trans-predicted and cis+trans-predicted analysis, respectively. Receiver Operating Characteristic (ROC) analysis of results from GSOR and TWAS are shown, respectively. TPR: true positive rate. FPR: false positive rate. hsq: heritability (0.1, 0.3, 0.5, 0.7 and 0.9) of simulated traits. The significance of the difference in the area under the curve between TWAS and GSOR was p=0.89 for cis-predicted, p=0.96 for trans-predicted and p=0.09 for cis+trans-predicted.


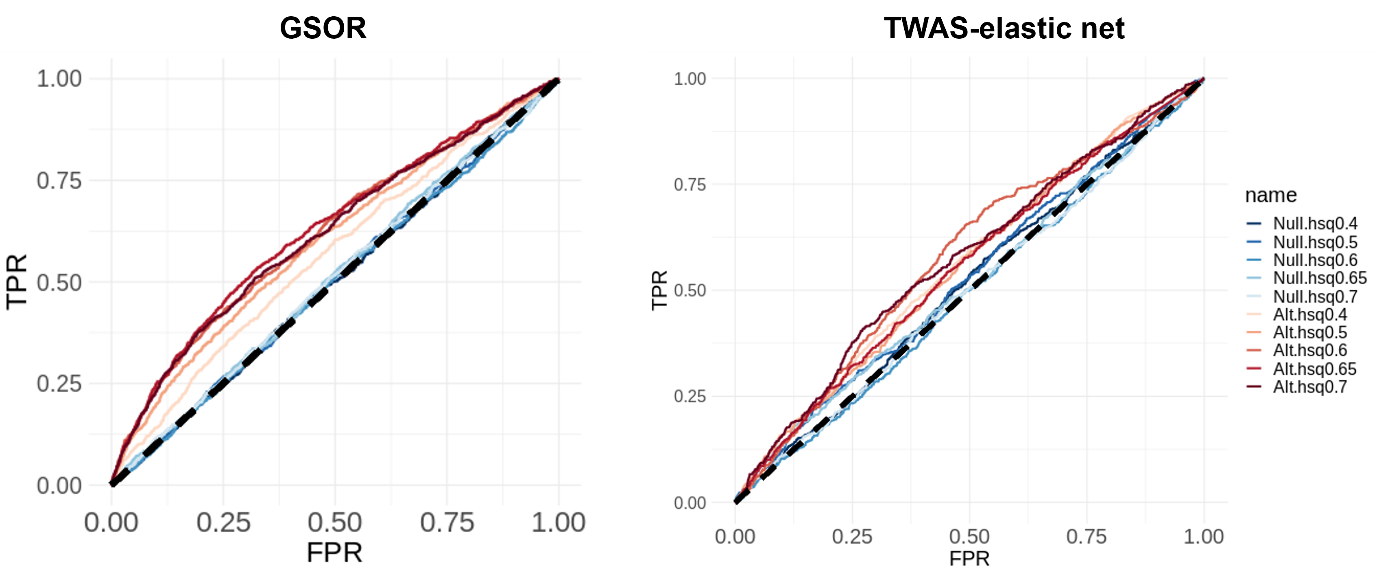


**Supplementary Figure 5**. Comparison of results between GSOR and TWAS (elastic net) using simulations. TWAS used the model of elastic net implemented in PrediXcan based on simulated data. TPR: true positive rate. FPR: false positive rate. hsq: heritability (0.4, 0.5, 0.6, 0.65 and 0.7) of simulated traits. The significance of the difference in the area under the curve between GSOR and TWAS (elastic net) averaged across scenarios is p = 0.02.


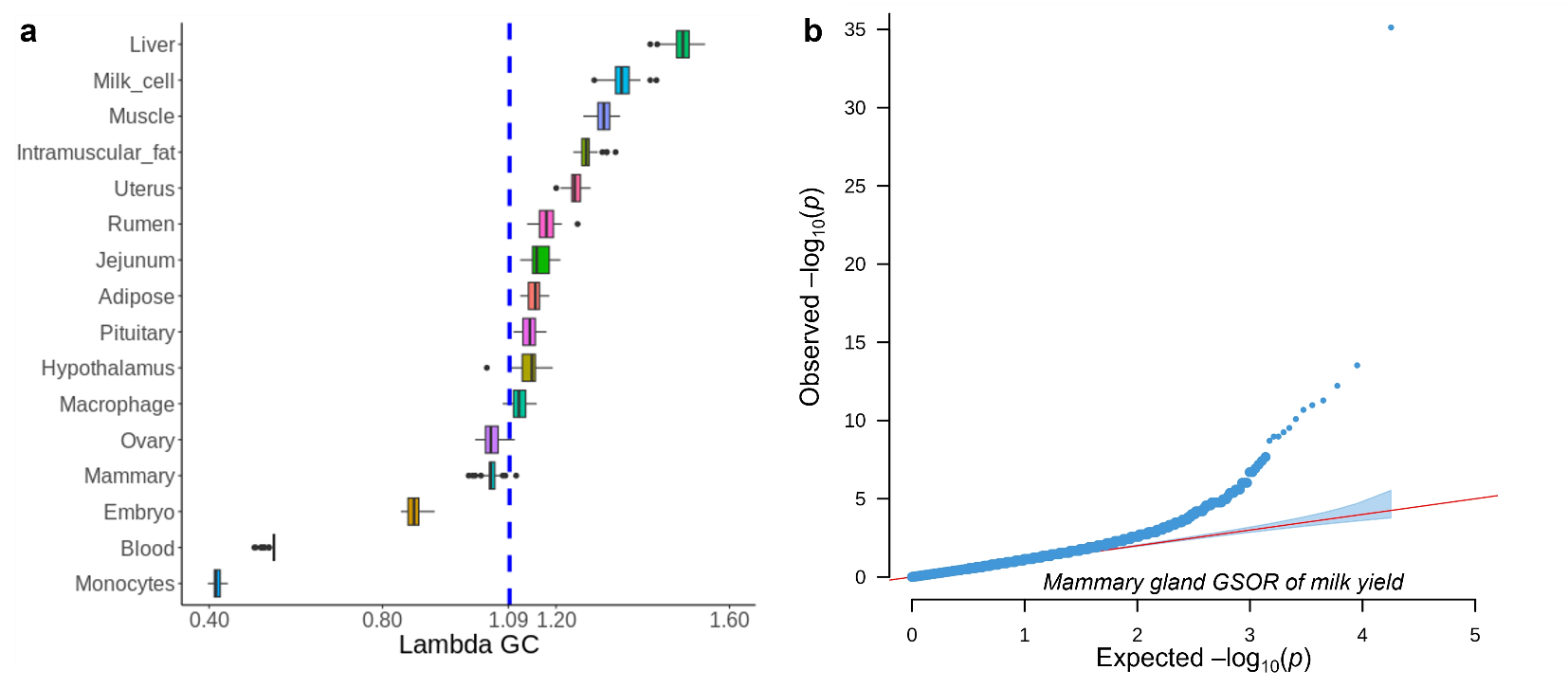


**Supplementary Figure 6**. Genome-wide inflation factor (lambda) for GSOR. **a**: lambda of GSOR across 16 tissues where each box represents lambda distribution across 37 traits. the blue dashed line indicates the mean of lambda across all studies which is 1.09. **b**: An example of the QQ plot of GSOR of mammary gland for milk yield with the lambda of 1.06.


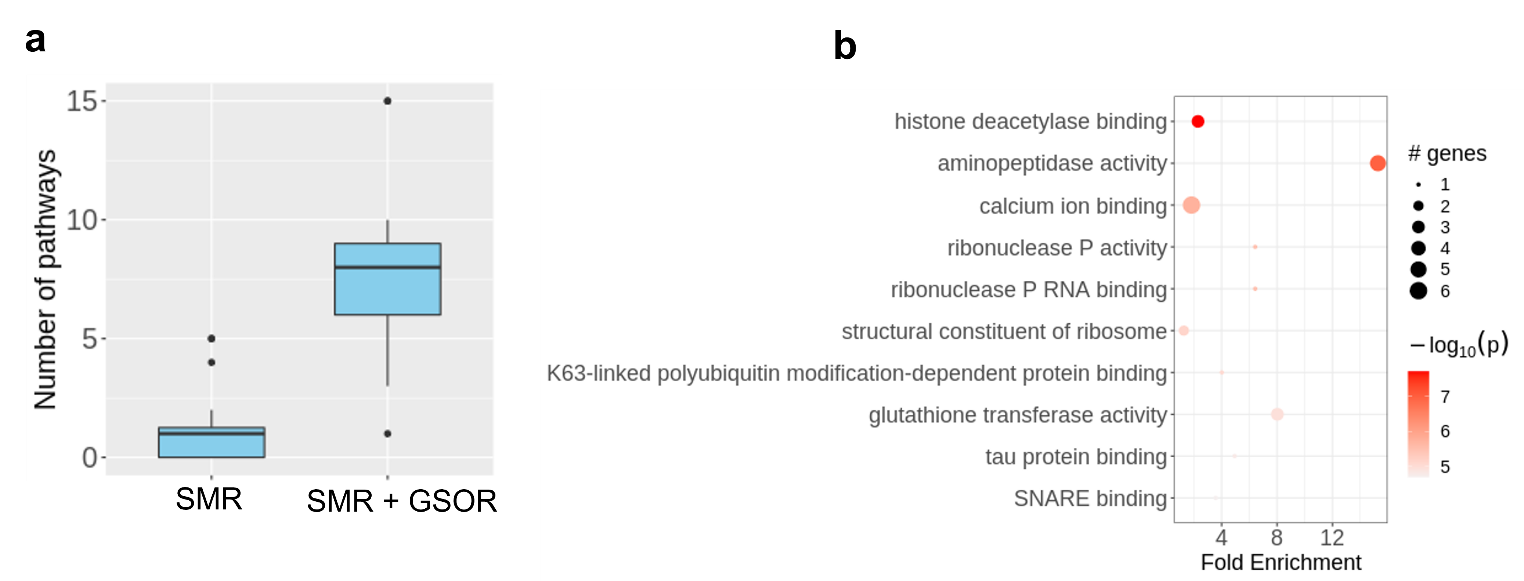


**Supplementary Figure 7**. Comparing GO term enrichment identified for the top 500 genes detected only by SMR and by both SMR and GSOR across tissues and traits. **a**: Comparing the number of pathways significantly enriched (adjusted p < 0.05) for the top 500 genes detected only by SMR with the top 500 genes detected by both SMR and GSOR. **b**: Enriched pathways for the top 500 genes detected by both SMR and GSOR in milk cells. These pathways are not enriched for genes detected only by SMR. On average, the number of genes overlapped between the two methods is 143(SD=48); The number of pathways overlapped between the two methods <=1 for each tissue (Supplementary Table 9).


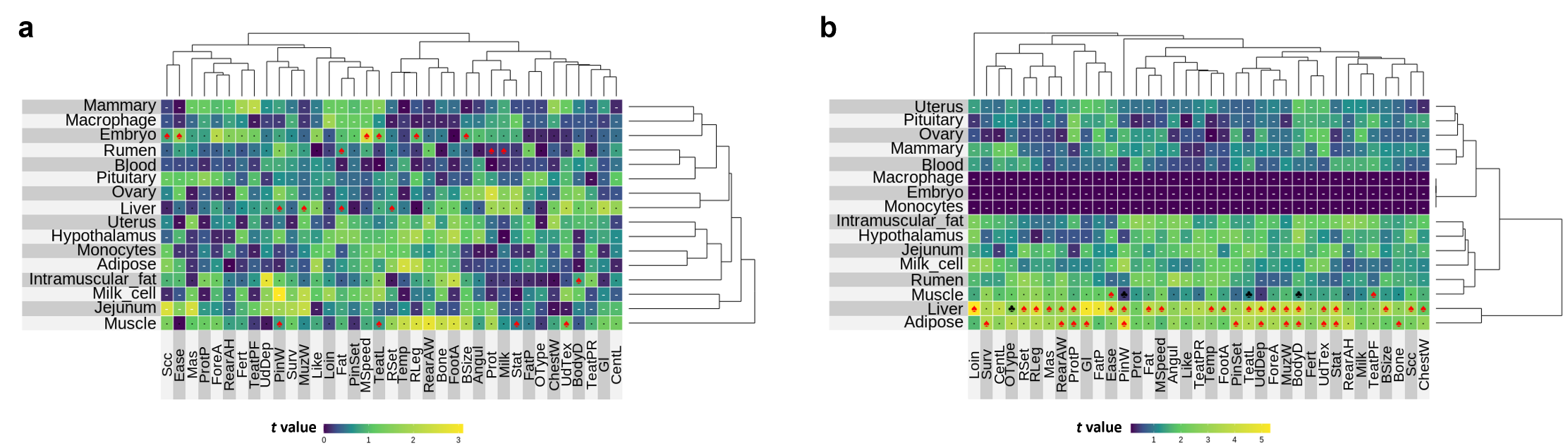


**Supplementary Figure 8**. The heat map of effects of IGF2 expression (**a**) and splicing (**b**) across tissues and traits based on GSOR. In these heat maps, red spades indicate regulatory effects interred using summary-based Mendelian randomisation (SMR) independent of LD; black hearts indicate the regulatory effects confounded by LD while black clubs indicate regulatory effects without testing LD due to not enough SNPs. Black dots represent insignificant SMR test and white hyphens indicate no e/sQTL or QTL can be used for SMR test. The dendrogram represents the hierarchical clustering of effects. The color scale of heatmaps is based on the magnitude of t (beta/SE) value of GSOR.


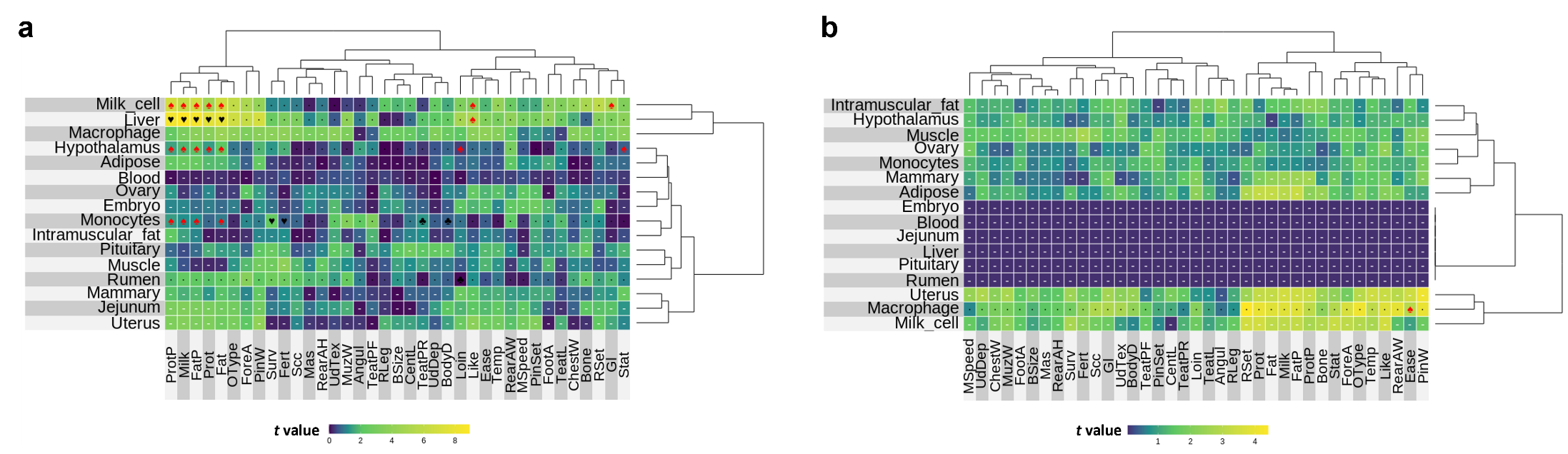


**Supplementary Figure 9**. The heat map of effects of *MGST1* expression (**a**) and splicing (**b**) across tissues and traits based on GSOR. In these heat maps, red spades indicate regulatory effects interred using summary-based Mendelian randomisation (SMR) independent of LD; black hearts indicate the regulatory effects confounded by LD while black clubs indicate regulatory effects without testing LD due to not enough SNPs. Black dots represent insignificant SMR test and white hyphens indicate no e/sQTL or QTL can be used for SMR test. The dendrogram represents the hierarchical clustering of effects. The color scale of heatmaps is based on the magnitude of t (beta/SE) value of GSOR.


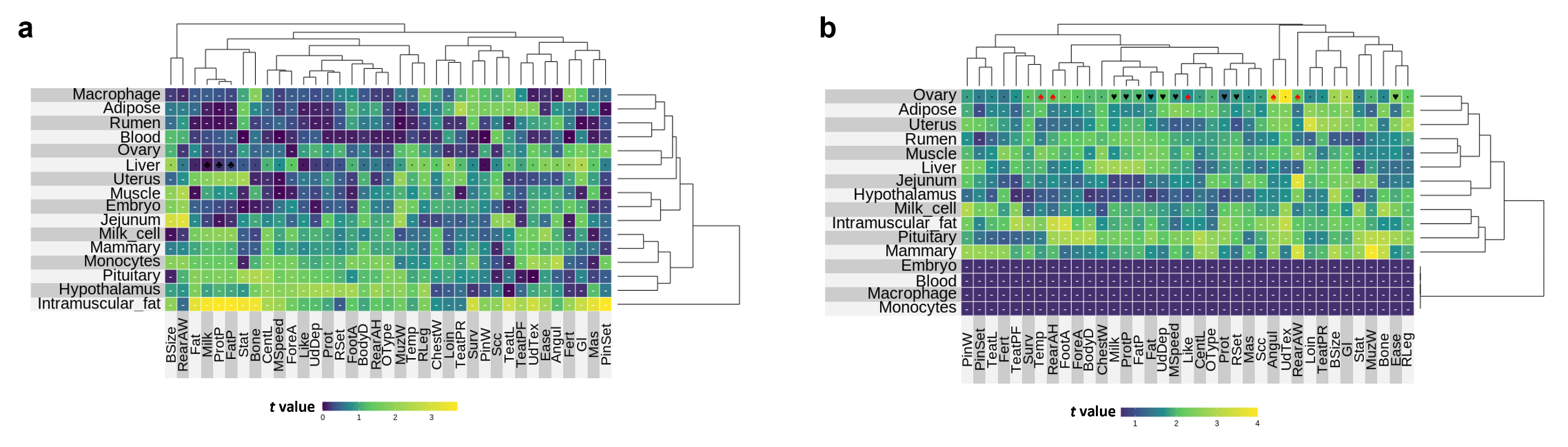


**Supplementary Figure 10**. The heat map of effects of *GHR* expression (**a**) and splicing (**b**) across tissues and traits based on GSOR. In these heat maps, red spades indicate regulatory effects interred using summary-based Mendelian randomisation (SMR) independent of LD; black hearts indicate the regulatory effects confounded by LD while black clubs indicate regulatory effects without testing LD due to not enough SNPs. Black dots represent insignificant SMR test and white hyphens indicate no e/sQTL or QTL can be used for SMR test. The dendrogram represents the hierarchical clustering of effects. The color scale of heatmaps is based on the magnitude of t (beta/SE) value of GSOR.

| **Supplementary Table 1**. Cattle traits analysed in the study.cow.N: number of cows for each trait. bull.N: number of bulls for each trait. | | | | |
| --- | --- | --- | --- | --- |
| trait full name | short.name | trait.order | cow.N | bull.N |
| protein yield | Prot | tr01 | 76659 | 8097 |
| fat yield | Fat | tr02 | 76659 | 8097 |
| milk yield | Milk | tr03 | 76659 | 8097 |
| protein percentage | ProtP | tr04 | 76659 | 8097 |
| fat percentage | FatP | tr05 | 76659 | 8097 |
| mastitis | Mas | tr06 | 77642 | 8103 |
| somatic cell count | Scc | tr07 | 75429 | 8083 |
| survival | Surv | tr08 | 61056 | 7147 |
| fertility | Fert | tr09 | 56840 | 7254 |
| ease (of birth) | Ease | tr10 | 43970 | 7835 |
| birth size | BSize | tr11 | 43755 | 7827 |
| gestation length | Gl | tr12 | 37214 | 7181 |
| temperament | Temp | tr13 | 37355 | 6966 |
| milking speed | MSpeed | tr14 | 37303 | 6966 |
| likeability | Like | tr15 | 37323 | 6966 |
| stature | Stat | tr16 | 45056 | 7022 |
| chest width | ChestW | tr17 | 45056 | 7022 |
| angularity | Angul | tr18 | 45056 | 7022 |
| bone quality | Bone | tr19 | 45056 | 7022 |
| rear legs set | RSet | tr20 | 45056 | 7022 |
| fore attachment | ForeA | tr21 | 45056 | 7022 |
| rear attachment height | RearAH | tr22 | 45056 | 7022 |
| front teat placement | TeatPF | tr23 | 45056 | 7022 |
| loin strength | Loin | tr24 | 45056 | 7022 |
| udder texture | UdTex | tr25 | 45056 | 7022 |
| central ligament | CentL | tr26 | 45055 | 7022 |
| pin width | PinW | tr27 | 45055 | 7022 |
| foot angle | FootA | tr28 | 45055 | 7022 |
| udder depth | UdDep | tr29 | 45055 | 7022 |
| pin set | PinSet | tr30 | 45054 | 7022 |
| rear attachment width | RearAW | tr31 | 45054 | 7022 |
| muzzle width | MuzW | tr32 | 45054 | 7022 |
| teat length | TeatL | tr33 | 45053 | 7022 |
| body depth | BodyD | tr34 | 45052 | 7022 |
| overall type | OType | tr35 | 44930 | 7022 |
| rear teat placement | TeatPR | tr36 | 44706 | 7022 |
| rear leg view | RLeg | tr37 | 44685 | 7022 |

| **Supplementary Table 2**. The difference in the area under curves (AUC) between the analyses of GSOR and TWAS using simulated data (further analysis for Figure 2a,b and Supplementary Figure 1). gsor_twas.diff: difference in the mean of AUC between analyses of GSOR and TWAS across different simulation scenarios. gsor_twas.SE: standard error of gsor_twas.diff. gsor_twas.p: p-value of gsor_twas.diff. | | | |
| --- | --- | --- | --- |
| Type | gsor_twas.diff | gsor_twas.SE | gsor_twas.p |
| cis | 0.0543 | 0.0135 | 5.6E-05 |
| trans | -0.0026 | 0.0086 | 0.7660 |
| cis + trans | 0.0633 | 0.0126 | 5.4E-07 |

| **Supplementary Table 3**. Comparison of significant genes that are causal (sharing causal eQTL and QTL) in simulations detected by different methods. TWAS.GBLUP: TWAS using GBLUP to train the model. TWAS.ELnet: TWAS uses an elastic net (PrediXcan) to train the model. m.nGene.sig: average number of genes that are significant at p-value < 0.05 across simulated traits. SE.nGene.sig: standard error of m.nGene.sig. m.nGene.sigcau: average number of genes that are significant at p < 0.05 and are also causal genes. m.prop.sigcau: average proportion of genes that are significant at p < 0.05 and are also causal. SE.prop.sigcau: standard error of m.prop.sigcau. For m.prop.sigcau, the p-value of difference between GSOR and TWAS.BLUP is 0.015 and the p-value of the difference between GSOR and TWAS.ELnet is 0.001. The difference in m.prop.sigcau between TWAS.BLUP and TWAS.ELnet is not significant. | | | | | | |
| --- | --- | --- | --- | --- | --- | --- |
| Methods | m.nGene.sig | SE.nGene.sig | m.nGene.sigcau | SE.nGene.sigcau | m.prop.sigcau | SE.prop.sigcau |
| GSOR | 340 | 2.4 | 237 | 8.1 | 0.6977 | 0.0220 |
| TWAS.BLUP | 1190 | 33.1 | 737 | 53.6 | 0.6201 | 0.0230 |
| TWAS.ELnet | 1345 | 63.6 | 829 | 117.6 | 0.6171 | 0.0116 |

| **Supplementary Table 4**. The number of independent individuals per tissue used for eQTL and sQTL mapping. | | | |
| --- | --- | --- | --- |
| Tissue | N of individuals | Generation |  |
| Blood | 945 | AVR (356) + CattleGTEx (589) |  |
| Muscle | 699 | CattleGTEx |  |
| Liver | 576 | CattleGTEx |  |
| Uterus | 359 | CattleGTEx |  |
| Macrophage | 295 | CattleGTEx |  |
| Embryo | 281 | CattleGTEx |  |
| Milk_cell | 268 | AVR |  |
| Rumen | 202 | CattleGTEx |  |
| Mammary | 175 | CattleGTEx |  |
| Muscle (Cesar et al.) | 171 | CattleGTEx |  |
| Adipose | 151 | CattleGTEx |  |
| Ovary | 139 | CattleGTEx |  |
| Pituitary | 134 | CattleGTEx |  |
| Monocytes | 113 | CattleGTEx |  |
| Hypothalamus | 112 | CattleGTEx |  |
| Jejunum | 105 | CattleGTEx |  |
| Average | 295 |  |  |
| Total | 4725 |  |  |
| AVR: dataset generated by Agriculture Victoria, Australia. | | |  |
| CattleGTEx: data from the Cattle GTEx consortium ^68^. | | | |

| **Supplementary Table 5**. Summary of results of MTAO. tot.Ngene: total number of genes/intron analysed for each tissue. n.sig.Ngene: number of genes with nominal p-value of Pn (number of traits affected) < 0.05; ave.Pn: average value of Pn. pn.sig.padj.Ngene: number of genes multi-testing adjusted p-value of Pn < 0.05; ave.Pn.padj: average value of Pn using adjusted p-value cutoff. pm.sig.Ngene: number of genes with nominal p-value of Pm (magnitude of multi-trait effects) < 0.05; ave.Pm: average value of Pm. pm.sig.padj.Ngene: number of genes with multi-testing adjusted p-value of Pm < 0.05; ave.Pm.padj: average value of Pm using adjusted p-value cutoff. | | | | | | | | | | |
| --- | --- | --- | --- | --- | --- | --- | --- | --- | --- | --- |
| genetype | Tissue | tot.Ngene | pn.sig.Ngene | ave.Pn | pn.sig.padj.Ngene | ave.Pn.padj | pm.sig.Ngene | ave.Pm | pm.sig.padj.Ngene | ave.Pm.padj |
| gene | Adipose | 17660 | 3572 | 8.92 | 2769 | 10.06 | 5003 | 9.30 | 3856 | 9.85 |
|  | Blood | 16564 | 2853 | 12.82 | 2554 | 13.74 | 3469 | 13.35 | 3070 | 14.12 |
|  | Embryo | 16211 | 1992 | 8.27 | 1154 | 10.34 | 2950 | 8.89 | 1986 | 9.55 |
|  | Hypothalamus | 18628 | 3683 | 8.70 | 2854 | 9.78 | 5191 | 9.18 | 3962 | 9.71 |
|  | Intramuscular_fat | 16199 | 4170 | 9.81 | 3369 | 10.95 | 5574 | 9.76 | 4690 | 10.21 |
|  | Jejunum | 18897 | 4003 | 9.15 | 3136 | 10.30 | 5556 | 9.43 | 4353 | 9.97 |
|  | Liver | 16532 | 5629 | 11.18 | 4801 | 12.24 | 7009 | 11.01 | 6275 | 11.43 |
|  | Macrophage | 14770 | 2889 | 8.47 | 2211 | 9.53 | 4110 | 9.05 | 3098 | 9.57 |
|  | Mammary | 17966 | 3177 | 9.28 | 2523 | 10.39 | 4479 | 9.46 | 3385 | 10.10 |
|  | Milk_cell | 16286 | 4746 | 10.04 | 3907 | 11.12 | 6177 | 10.08 | 5350 | 10.49 |
|  | Monocytes | 16141 | 1504 | 9.40 | 965 | 11.64 | 2064 | 9.70 | 1469 | 10.56 |
|  | Muscle | 15856 | 4435 | 10.36 | 3678 | 11.46 | 5731 | 10.33 | 4917 | 10.81 |
|  | Ovary | 18180 | 3054 | 8.52 | 2377 | 9.53 | 4449 | 9.04 | 3266 | 9.60 |
|  | Pituitary | 18862 | 3721 | 8.68 | 2824 | 9.84 | 5235 | 9.22 | 4026 | 9.74 |
|  | Rumen | 17490 | 3793 | 9.45 | 2973 | 10.67 | 5140 | 9.63 | 4118 | 10.17 |
|  | Uterus | 18589 | 4567 | 9.79 | 3696 | 10.91 | 6109 | 9.82 | 5091 | 10.30 |
| intron | Adipose | 214602 | 29800 | 8.05 | 15925 | 10.34 | 45114 | 8.82 | 30074 | 9.48 |
|  | Blood | 171580 | 13405 | 11.23 | 9660 | 13.48 | 17497 | 11.61 | 13301 | 12.88 |
|  | Embryo | 30132 | 3140 | 7.67 | 1595 | 9.86 | 4944 | 8.63 | 3022 | 9.31 |
|  | Hypothalamus | 192856 | 27340 | 7.76 | 14476 | 9.84 | 41671 | 8.69 | 27841 | 9.28 |
|  | Intramuscular_fat | 125055 | 16685 | 8.39 | 9485 | 10.64 | 25506 | 8.92 | 17466 | 9.57 |
|  | Jejunum | 165578 | 22885 | 8.05 | 12483 | 10.23 | 34553 | 8.81 | 23262 | 9.44 |
|  | Liver | 196601 | 34262 | 8.92 | 26276 | 10.11 | 49214 | 9.33 | 36280 | 9.99 |
|  | Macrophage | 153138 | 17920 | 7.71 | 9360 | 9.82 | 27825 | 8.65 | 17471 | 9.31 |
|  | Mammary | 220783 | 18661 | 8.12 | 9992 | 10.47 | 29552 | 8.81 | 17108 | 9.70 |
|  | Milk_cell | 221343 | 49114 | 9.47 | 38991 | 10.63 | 66768 | 9.72 | 53388 | 10.29 |
|  | Monocytes | 146270 | 10290 | 7.47 | 5032 | 9.64 | 16666 | 8.55 | 8906 | 9.38 |
|  | Muscle | 172523 | 29626 | 8.64 | 22382 | 9.82 | 42830 | 9.16 | 31149 | 9.79 |
|  | Ovary | 177820 | 25057 | 7.86 | 13451 | 9.96 | 37725 | 8.74 | 25602 | 9.33 |
|  | Pituitary | 206952 | 27829 | 7.79 | 14512 | 9.97 | 42671 | 8.71 | 27705 | 9.35 |
|  | Rumen | 175348 | 22048 | 8.04 | 11971 | 10.25 | 33958 | 8.79 | 22097 | 9.47 |
|  | Uterus | 212765 | 27569 | 8.34 | 15260 | 10.70 | 41148 | 8.97 | 27230 | 9.71 |

| **Supplementary Table 6**. Overlap of prioritised genes/introns between multi-trait meta-analysis of omics-associations (MTAO) and multi-trait summary data-based Mendelian randomization (SMR). N.total: total number of genes or introns testable between MTAO and SMR. N.mtaosig: Number of genes significant in MTAO. N.smrsig: number of genes significant in multi-trait SMR. N.smrsig.mtaosig: Number of genes significant in both MTAO and multi-trait SMR. odds.ratio: fisher's exact test on a contingency table based on N.smrsig.mtaosig, N.mtaosig-N.smrsig.mtaosig, N.smrsig-N.smrsig.mtaosig and N.total-N.mtaosig-N.smrsig +N.smrsig.mtaosig. p.fe.adj: FDR adjusted p-value of fisher's exact test. | | | | | | | |
| --- | --- | --- | --- | --- | --- | --- | --- |
| Omics feature | Tissue | N.total | N.mtaosig | N.smrsig | N.smrsig.mtaosig | odds.ratio | p.fe.adj |
| gene expression | Adipose | 1245 | 642 | 65 | 44 | 2.04 | 3.77E-02 |
|  | Blood | 6346 | 2390 | 490 | 276 | 2.28 | 3.36E-17 |
|  | Embryo | 2557 | 404 | 110 | 22 | 1.35 | 2.73E-01 |
|  | Hypothalamus | 2181 | 534 | 83 | 24 | 1.27 | 2.73E-01 |
|  | Intramuscular_fat | 2165 | 1061 | 93 | 59 | 1.85 | 2.67E-02 |
|  | Jejunum | 1012 | 542 | 49 | 35 | 2.25 | 4.17E-02 |
|  | Liver | 5344 | 2991 | 334 | 251 | 2.50 | 7.68E-13 |
|  | Macrophage | 1799 | 449 | 52 | 19 | 1.76 | 1.34E-01 |
|  | Mammary | 1286 | 699 | 75 | 69 | 10.59 | 4.13E-12 |
|  | Milk_cell | 1524 | 1405 | 133 | 130 | 3.94 | 3.77E-02 |
|  | Monocytes | 4866 | 593 | 105 | 28 | 2.70 | 3.50E-04 |
|  | Muscle | 4597 | 2095 | 259 | 170 | 2.39 | 2.34E-10 |
|  | Ovary | 1986 | 361 | 78 | 21 | 1.70 | 1.34E-01 |
|  | Pituitary | 3481 | 1019 | 183 | 66 | 1.39 | 1.24E-01 |
|  | Rumen | 2937 | 1022 | 134 | 77 | 2.66 | 4.12E-07 |
|  | Uterus | 2987 | 1494 | 147 | 103 | 2.44 | 3.81E-06 |
| RNA splicing | Adipose | 9600 | 2377 | 430 | 173 | 2.13 | 3.77E-12 |
|  | Blood | 21453 | 6785 | 1571 | 795 | 2.38 | 3.34E-58 |
|  | Embryo | 2030 | 243 | 60 | 17 | 3.05 | 1.20E-03 |
|  | Hypothalamus | 9505 | 1450 | 340 | 73 | 1.55 | 2.34E-03 |
|  | Intramuscular_fat | 11656 | 2396 | 489 | 157 | 1.89 | 4.33E-09 |
|  | Jejunum | 7259 | 1372 | 278 | 95 | 2.32 | 4.33E-09 |
|  | Liver | 19824 | 6319 | 986 | 434 | 1.73 | 2.15E-15 |
|  | Macrophage | 14543 | 1899 | 571 | 96 | 1.36 | 4.99E-03 |
|  | Mammary | 10870 | 2074 | 336 | 142 | 3.26 | 3.45E-22 |
|  | Milk_cell | 8955 | 7786 | 798 | 747 | 2.33 | 1.49E-09 |
|  | Monocytes | 9571 | 751 | 296 | 42 | 2.00 | 5.75E-04 |
|  | Muscle | 18118 | 5108 | 923 | 383 | 1.87 | 4.95E-18 |
|  | Ovary | 14326 | 2159 | 631 | 129 | 1.48 | 5.75E-04 |
|  | Pituitary | 15620 | 2644 | 609 | 142 | 1.52 | 1.39E-04 |
|  | Rumen | 13010 | 2199 | 459 | 133 | 2.07 | 4.15E-10 |
|  | Uterus | 14658 | 3778 | 602 | 296 | 2.94 | 3.56E-35 |

| **Supplementary Table 7**. cis eQTL used to test SMR and HEIDI for DGAT1 and its neighbour genes of ZNF34 and IQANK1. N number of SNPs. | | | |
| --- | --- | --- | --- |
| N of eQTL for DGAT1 | N of eQTL for ZNF34 | N of eQTL for both DGAT1 and ZNF34 | average LD-r between eQTL of DGAT1 and eQTL of ZNF34 |
| 1554 | 1341 | 463 | 0.707 |
| N of eQTL for DGAT1 | N of eQTL for IQANK1 | N of eQTL for both DGAT1 and IQANK1 | average LD-r between eQTL of DGAT1 and eQTL of IQANK1 |
| 1554 | 662 | 317 | 0.708 |

**Supplementary Table 8**. Comparison of genes and pathways for SMR and SMR + GSOR. nGene_ovlp: the number of genes that overlap between the two methods (the top 500 genes were selected from each method). nPathways: the number of significant pathways detected by each method. nPathways_ovlp: the number of pathways that overlap between the two methods.

| Tissue | Analysis | nGene_ovlp | nPathways | nPathways_ovlp |
| --- | --- | --- | --- | --- |
| Adipose | SMR | 130 | 0 | 0 |
| Adipose | SMR+GSOR | 130 | 7 | 0 |
| Blood | SMR | 98 | 3 | 1 |
| Blood | SMR+GSOR | 98 | 4 | 1 |
| Embryo | SMR | 220 | 0 | 0 |
| Embryo | SMR+GSOR | 220 | 14 | 0 |
| Hypothalamus | SMR | 222 | 1 | 0 |
| Hypothalamus | SMR+GSOR | 222 | 8 | 0 |
| Intramuscular_fat | SMR | 86 | 0 | 0 |
| Intramuscular_fat | SMR+GSOR | 86 | 8 | 0 |
| Jejunum | SMR | 116 | 0 | 0 |
| Jejunum | SMR+GSOR | 116 | 5 | 0 |
| Liver | SMR | 95 | 3 | 1 |
| Liver | SMR+GSOR | 95 | 1 | 1 |
| Macrophage | SMR | 152 | 0 | 0 |
| Macrophage | SMR+GSOR | 152 | 13 | 0 |
| Mammary | SMR | 142 | 0 | 0 |
| Mammary | SMR+GSOR | 142 | 6 | 0 |
| Milk_cell | SMR | 134 | 0 | 0 |
| Milk_cell | SMR+GSOR | 134 | 7 | 0 |
| Monocytes | SMR | 213 | 1 | 1 |
| Monocytes | SMR+GSOR | 213 | 8 | 1 |
| Muscle | SMR | 98 | 1 | 0 |
| Muscle | SMR+GSOR | 98 | 9 | 0 |
| Ovary | SMR | 195 | 0 | 0 |
| Ovary | SMR+GSOR | 195 | 4 | 0 |
| Pituitary | SMR | 148 | 5 | 0 |
| Pituitary | SMR+GSOR | 148 | 4 | 0 |
| Rumen | SMR | 94 | 3 | 1 |
| Rumen | SMR+GSOR | 94 | 4 | 1 |
| Uterus | SMR | 107 | 0 | 0 |
| Uterus | SMR+GSOR | 107 | 10 | 0 |

| **Supplementary Table 9**. correlation between tissue ranking and sample size. rho: spearman correlation coefficient; p: raw p-value of rho; p.adjust: FDR adjusted p.value. | | | | |
| --- | --- | --- | --- | --- |
| type | tr | rho | p | p.adj |
| gene | Prot | 0.188235 | 0.483876 | 0.628522 |
|  | Fat | 0.273529 | 0.304279 | 0.50296 |
|  | Milk | 0.382353 | 0.14467 | 0.454856 |
|  | ProtP | 0.4 | 0.125879 | 0.454856 |
|  | FatP | 0.452941 | 0.080004 | 0.426002 |
|  | Mas | 0.173529 | 0.519358 | 0.628522 |
|  | Scc | 0.15 | 0.57858 | 0.648711 |
|  | Surv | 0.344118 | 0.191942 | 0.454856 |
|  | Fert | 0.344118 | 0.191942 | 0.454856 |
|  | Ease | 0.241176 | 0.366896 | 0.543006 |
|  | BSize | 0.491176 | 0.05558 | 0.411294 |
|  | Gl | 0.529412 | 0.037277 | 0.379379 |
|  | Temp | 0.529412 | 0.037277 | 0.379379 |
|  | MSpeed | 0.261765 | 0.326245 | 0.50296 |
|  | Like | 0.102941 | 0.704933 | 0.745215 |
|  | Stat | 0.45 | 0.082167 | 0.426002 |
|  | ChestW | 0.261765 | 0.326245 | 0.50296 |
|  | Angul | 0.170588 | 0.526599 | 0.628522 |
|  | Bone | 0.329412 | 0.212614 | 0.454856 |
|  | RSet | 0.305882 | 0.248661 | 0.484235 |
|  | ForeA | 0.117647 | 0.664435 | 0.723061 |
|  | RearAH | -0.02059 | 0.943224 | 0.943224 |
|  | TeatPF | 0.173529 | 0.519358 | 0.628522 |
|  | Loin | 0.061765 | 0.822182 | 0.84502 |
|  | UdTex | 0.335294 | 0.204176 | 0.454856 |
|  | CentL | 0.267647 | 0.315146 | 0.50296 |
|  | PinW | 0.158824 | 0.556032 | 0.642912 |
|  | FootA | 0.361765 | 0.168981 | 0.454856 |
|  | UdDep | 0.423529 | 0.103622 | 0.426002 |
|  | PinSet | 0.323529 | 0.221281 | 0.454856 |
|  | RearAW | 0.641176 | 0.008975 | 0.332092 |
|  | MuzW | 0.426471 | 0.101056 | 0.426002 |
|  | TeatL | 0.282353 | 0.288413 | 0.50296 |
|  | BodyD | 0.335294 | 0.204176 | 0.454856 |
|  | OType | 0.223529 | 0.403941 | 0.562275 |
|  | TeatPR | 0.220588 | 0.410309 | 0.562275 |
|  | RLeg | 0.520588 | 0.041014 | 0.379379 |
| intron | Prot | 0.323529 | 0.221281 | 0.340547 |
|  | Fat | 0.25 | 0.349132 | 0.430596 |
|  | Milk | 0.441176 | 0.088915 | 0.205617 |
|  | ProtP | 0.45 | 0.082167 | 0.202678 |
|  | FatP | 0.452941 | 0.080004 | 0.202678 |
|  | Mas | 0.488235 | 0.057228 | 0.186955 |
|  | Scc | 0.314706 | 0.234712 | 0.340547 |
|  | Surv | 0.582353 | 0.02004 | 0.151279 |
|  | Fert | 0.714706 | 0.002588 | 0.095772 |
|  | Ease | 0.482353 | 0.060634 | 0.186955 |
|  | BSize | 0.388235 | 0.1382 | 0.269126 |
|  | Gl | 0.311765 | 0.239304 | 0.340547 |
|  | Temp | 0.105882 | 0.696767 | 0.696767 |
|  | MSpeed | 0.208824 | 0.436322 | 0.461255 |
|  | Like | 0.338235 | 0.200041 | 0.321805 |
|  | Stat | 0.194118 | 0.470031 | 0.483088 |
|  | ChestW | 0.364706 | 0.165346 | 0.304227 |
|  | Angul | 0.25 | 0.349132 | 0.430596 |
|  | Bone | 0.660714 | 0.009043 | 0.151279 |
|  | RSet | 0.35 | 0.184066 | 0.309566 |
|  | ForeA | 0.491176 | 0.05558 | 0.186955 |
|  | RearAH | 0.358824 | 0.17267 | 0.304227 |
|  | TeatPF | 0.408824 | 0.117166 | 0.252679 |
|  | Loin | 0.273529 | 0.304279 | 0.402083 |
|  | UdTex | 0.6 | 0.01597 | 0.151279 |
|  | CentL | 0.244118 | 0.360918 | 0.430773 |
|  | PinW | 0.402941 | 0.122925 | 0.252679 |
|  | FootA | 0.461765 | 0.073768 | 0.202678 |
|  | UdDep | 0.291176 | 0.27307 | 0.374207 |
|  | PinSet | 0.226471 | 0.397628 | 0.453502 |
|  | RearAW | 0.547059 | 0.030594 | 0.151279 |
|  | MuzW | 0.217647 | 0.416731 | 0.453502 |
|  | TeatL | 0.217647 | 0.416731 | 0.453502 |
|  | BodyD | 0.541176 | 0.032709 | 0.151279 |
|  | OType | 0.561765 | 0.025771 | 0.151279 |
|  | TeatPR | 0.525 | 0.047104 | 0.186955 |
|  | RLeg | 0.552941 | 0.028588 | 0.151279 |

**References**:

1 Gusev, A. *et al.* Integrative approaches for large-scale transcriptome-wide association studies. *Nature genetics* **48**, 245-252 (2016).

2 Gamazon, E. R. *et al.* A gene-based association method for mapping traits using reference transcriptome data. *Nature genetics* **47**, 1091-1098 (2015).

3 Wray, N. R., Kemper, K. E., Hayes, B. J., Goddard, M. E. & Visscher, P. M. Complex Trait Prediction from Genome Data: Contrasting EBV in Livestock to PRS in Humans: Genomic Prediction. *Genetics* **211**, 1131-1141 (2019).

4 Xiang, R. *et al.* Genome-wide fine-mapping identifies pleiotropic and functional variants that predict many traits across global cattle populations. *Nature communications* **12**, 1-13 (2021).

5 Kemper, K. E. *et al.* Improved precision of QTL mapping using a nonlinear Bayesian method in a multi-breed population leads to greater accuracy of across-breed genomic predictions. *Genetics Selection Evolution* **47**, 29 (2015).

6 Bolormaa, S. *et al.* A Multi-Trait, Meta-analysis for Detecting Pleiotropic Polymorphisms for Stature, Fatness and Reproduction in Beef Cattle. *PLOS Genetics* **10**, e1004198, doi:10.1371/journal.pgen.1004198 (2014).

7 Consortium, G. Genetic effects on gene expression across human tissues. *Nature* **550**, 204 (2017).

8 Li, Y. I. *et al.* RNA splicing is a primary link between genetic variation and disease. *Science (New York, N.Y.)* **352**, 600-604, doi:10.1126/science.aad9417 (2016).

9 Grisart, B. *et al.* Positional candidate cloning of a QTL in dairy cattle: identification of a missense mutation in the bovine DGAT1 gene with major effect on milk yield and composition. *Genome research* **12**, 222-231 (2002).

10 Fink, T. *et al.* A new mechanism for a familiar mutation–bovine DGAT1 K232A modulates gene expression through multi-junction exon splice enhancement. *BMC genomics* **21**, 1-13 (2020).

11 Grisart, B. *et al.* Genetic and functional confirmation of the causality of the DGAT1 K232A quantitative trait nucleotide in affecting milk yield and composition. *Proceedings of the National Academy of Sciences* **101**, 2398-2403 (2004).

12 Fürbass, R., Winter, A., Fries, R. & Kuhn, C. Alleles of the bovine DGAT1 variable number of tandem repeat associated with a milk fat QTL at chromosome 14 can stimulate gene expression. *Physiological genomics* **25**, 116-120 (2006).

13 Kühn, C. *et al.* Evidence for multiple alleles at the DGAT1 locus better explains a quantitative trait locus with major effect on milk fat content in cattle. *Genetics* **167**, 1873-1881 (2004).

14 Liu, S. *et al.* A comprehensive catalogue of regulatory variants in the cattle transcriptome. *bioRxiv*, 2020.2012.2001.406280, doi:10.1101/2020.12.01.406280 (2021).

15 Clark, E. L. *et al.* From FAANG to fork: application of highly annotated genomes to improve farmed animal production. *Genome Biology* **21**, 1-9 (2020).

16 Zhu, Z. *et al.* Integration of summary data from GWAS and eQTL studies predicts complex trait gene targets. *Nature genetics* **48**, 481-487 (2016).

17 Kryazhimskiy, S. & Plotkin, J. B. The population genetics of dN/dS. *PLoS genetics* **4**, e1000304 (2008).

18 Storey, J. D. & Tibshirani, R. Statistical significance for genomewide studies. *Proceedings of the National Academy of Sciences* **100**, 9440-9445 (2003).

19 Jordan, D. M., Verbanck, M. & Do, R. HOPS: a quantitative score reveals pervasive horizontal pleiotropy in human genetic variation is driven by extreme polygenicity of human traits and diseases. *Genome Biology* **20**, 222, doi:10.1186/s13059-019-1844-7 (2019).

20 Xiang, R. *et al.* Mutant alleles differentially shape fitness and other complex traits in cattle. *Communications Biology* **4**, 1-10 (2021).

21 Xiang, R. *et al.* Gene expression and RNA splicing explain large proportions of the heritability for complex traits in cattle. *bioRxiv* (2022).

22 Wu, Y. *et al.* Integrative analysis of omics summary data reveals putative mechanisms underlying complex traits. *Nature communications* **9**, 1-14 (2018).

23 Wu, Y. *et al.* Promoter-anchored chromatin interactions predicted from genetic analysis of epigenomic data. *Nature communications* **11**, 1-12 (2020).

24 Van Laere, A.-S. *et al.* A regulatory mutation in IGF2 causes a major QTL effect on muscle growth in the pig. *Nature* **425**, 832-836 (2003).

25 Littlejohn, M. D. *et al.* Sequence-based Association Analysis Reveals an MGST1 eQTL with Pleiotropic Effects on Bovine Milk Composition. *Scientific Reports* **6**, 25376, doi:10.1038/srep25376

<https://www.nature.com/articles/srep25376#supplementary-information> (2016).

26 Viitala, S. *et al.* The role of the bovine growth hormone receptor and prolactin receptor genes in milk, fat and protein production in Finnish Ayrshire dairy cattle. *Genetics* **173**, 2151-2164 (2006).

27 Xiang, R. *et al.* Quantifying the contribution of sequence variants with regulatory and evolutionary significance to 34 bovine complex traits. *Proceedings of the National Academy of Sciences* **116**, 19398-19408 (2019).

28 Lee, S.-K., Choi, H.-J. & Ahnn, J. Dicarbonyl/l-xylulose reductase (DCXR): The multifunctional pentosuria enzyme. *The international journal of biochemistry & cell biology* **45**, 2563-2567 (2013).

29 Campbell, H. D. *et al.* Fliih, a gelsolin-related cytoskeletal regulator essential for early mammalian embryonic development. *Molecular and cellular biology* **22**, 3518-3526 (2002).

30 Li, Y. I. *et al.* Annotation-free quantification of RNA splicing using LeafCutter. *Nature genetics* **50**, 151 (2018).

31 Hormozdiari, F. *et al.* Colocalization of GWAS and eQTL signals detects target genes. *The American Journal of Human Genetics* **99**, 1245-1260 (2016).

32 Wen, X., Pique-Regi, R. & Luca, F. Integrating molecular QTL data into genome-wide genetic association analysis: Probabilistic assessment of enrichment and colocalization. *PLoS genetics* **13**, e1006646 (2017).

33 Giambartolomei, C. *et al.* Bayesian test for colocalisation between pairs of genetic association studies using summary statistics. *PLoS genetics* **10**, e1004383 (2014).

34 Rohart, F., Gautier, B., Singh, A. & Le Cao, K.-A. mixOmics: An R package for ‘omics feature selection and multiple data integration. *PLoS Computational Biology* **13**, e1005752 (2017).

35 Xiang, R., van den Berg, I., MacLeod, I. M., Daetwyler, H. D. & Goddard, M. E. Effect direction meta-analysis of GWAS identifies extreme, prevalent and shared pleiotropy in a large mammal. *Communications biology* **3**, 1-14 (2020).

36 Prowse-Wilkins, C. P. *et al.* Putative causal variants are enriched in annotated functional regions from six bovine tissues. *Frontiers in genetics* **12** (2021).

37 Xiang, R. *et al.* Genome variants associated with RNA splicing variations in bovine are extensively shared between tissues. *BMC genomics* **19**, 1-18 (2018).

38 Xiang, R., MacLeod, I. M., Bolormaa, S. & Goddard, M. E. Genome-wide comparative analyses of correlated and uncorrelated phenotypes identify major pleiotropic variants in dairy cattle. *Scientific Reports* **7**, 9248 (2017).

39 Abell, N. S. *et al.* Multiple causal variants underlie genetic associations in humans. *Science (New York, N.Y.)* **375**, 1247-1254 (2022).

40 Yang, H. *et al.* ABO genotype alters the gut microbiota by regulating GalNAc levels in pigs. *Nature*, 1-12 (2022).

41 Tiplady, K. M. *et al.* Sequence-based genome-wide association study of individual milk mid-infrared wavenumbers in mixed-breed dairy cattle. *Genetics Selection Evolution* **53**, 1-24 (2021).

42 Erbe, M. *et al.* Improving accuracy of genomic predictions within and between dairy cattle breeds with imputed high-density single nucleotide polymorphism panels. *Journal of dairy science* **95**, 4114-4129 (2012).

43 Breen, E. J. *et al.* BayesR3 enables fast MCMC blocked processing for largescale multi-trait genomic prediction and QTN mapping analysis. *Communications Biology* **5**, 661 (2022).

44 Daetwyler, H. *et al.* in *Proc Assoc Adv Anim Breed Genet.* 201-204.

45 Daetwyler, H. *et al.* Integration of functional genomics and phenomics into genomic prediction raises its accuracy in sheep and dairy cattle. *Proceedings of the Association for the Advancement of Animal Breeding and Genetics, Armidale, NSW, Australia*, 11-14 (2019).

46 Xiang, R. *et al.* Mutant alleles differentially shape cattle complex traits and fitness. *bioRxiv*, 2021.2004.2019.440546, doi:10.1101/2021.04.19.440546 (2021).

47 Clark, S. A. & van der Werf, J. in *Genome-Wide Association Studies and Genomic Prediction* 321-330 (Springer, 2013).

48 Maier, R. *et al.* Joint analysis of psychiatric disorders increases accuracy of risk prediction for schizophrenia, bipolar disorder, and major depressive disorder. *The American Journal of Human Genetics* **96**, 283-294 (2015).

49 Moser, G. *et al.* Simultaneous discovery, estimation and prediction analysis of complex traits using a Bayesian mixture model. *PLoS genetics* **11**, e1004969 (2015).

50 Zhang, F. *et al.* OSCA: a tool for omic-data-based complex trait analysis. *Genome biology* **20**, 1-13 (2019).

51 Hayes, B. J. & Daetwyler, H. D. 1000 Bull Genomes Project to Map Simple and Complex Genetic Traits in Cattle: Applications and Outcomes. *Annual review of animal biosciences* **7**, 89-102, doi:10.1146/annurev-animal-020518-115024 (2019).

52 Daetwyler, H. D. *et al.* Whole-genome sequencing of 234 bulls facilitates mapping of monogenic and complex traits in cattle. *Nature genetics* **46**, 858 (2014).

53 Yang, J., Lee, S. H., Goddard, M. E. & Visscher, P. M. GCTA: a tool for genome-wide complex trait analysis. *The American Journal of Human Genetics* **88**, 76-82 (2011).

54 Xiang, R. *et al.* Genome variants associated with RNA splicing variations in bovine are extensively shared between tissues. *BMC Genomics* **19**, 521, doi:10.1186/s12864-018-4902-8 (2018).

55 Chamberlain, A. *et al.* in *11th world congress on genetics applied to livestock production (WCGALP). Auckland, New Zealand: Volume Molecular Genetics.* 254.

56 Dobin, A. *et al.* STAR: ultrafast universal RNA-seq aligner. *Bioinformatics (Oxford, England)* **29**, 15-21, doi:10.1093/bioinformatics/bts635 (2013).

57 Rosen, B. D. *et al.* De novo assembly of the cattle reference genome with single-molecule sequencing. *GigaScience* **9**, giaa021 (2020).

58 Liao, Y., Smyth, G. K. & Shi, W. featureCounts: an efficient general purpose program for assigning sequence reads to genomic features. *Bioinformatics (Oxford, England)* **30**, 923-930 (2014).

59 Law, C. W., Chen, Y., Shi, W. & Smyth, G. K. Voom: precision weights unlock linear model analysis tools for RNA-seq read counts. *Genome Biology* **15**, R29 (2014).

60 Fuchsberger, C., Abecasis, G. R. & Hinds, D. A. minimac2: faster genotype imputation. *Bioinformatics (Oxford, England)* **31**, 782-784 (2014).

61 Howie, B., Fuchsberger, C., Stephens, M., Marchini, J. & Abecasis, G. R. Fast and accurate genotype imputation in genome-wide association studies through pre-phasing. *Nature genetics* **44**, 955 (2012).

62 Hayes, B. J., Visscher, P. M. & Goddard, M. E. Increased accuracy of artificial selection by using the realized relationship matrix. *Genetics research* **91**, 47-60 (2009).

63 Xiang, R. *et al.* Genome-wide fine-mapping identifies pleiotropic and functional variants that predict many traits across global cattle populations. *Nature Communications* **12**, 860, doi:10.1038/s41467-021-21001-0 (2021).

64 Xiang, R., van den Berg, I., MacLeod, I. M., Daetwyler, H. D. & Goddard, M. E. Effect direction meta-analysis of GWAS identifies extreme, prevalent and shared pleiotropy in a large mammal. *Commun Biol* **3**, 88, doi:10.1038/s42003-020-0823-6 (2020).

65 Stegle, O., Parts, L., Piipari, M., Winn, J. & Durbin, R. Using probabilistic estimation of expression residuals (PEER) to obtain increased power and interpretability of gene expression analyses. *Nature protocols* **7**, 500-507 (2012).

66 Verbanck, M., Chen, C.-Y., Neale, B. & Do, R. Detection of widespread horizontal pleiotropy in causal relationships inferred from Mendelian randomization between complex traits and diseases. *Nature genetics* **50**, 693-698 (2018).

67 Ulgen, E., Ozisik, O. & Sezerman, O. U. pathfindR: an R package for comprehensive identification of enriched pathways in omics data through active subnetworks. *Frontiers in genetics* **10**, 858 (2019).

68 Liu, S. *et al.* A multi-tissue atlas of regulatory variants in cattle. *Nature Genetics* **54**, 1438-1447, doi:10.1038/s41588-022-01153-5 (2022).
